# Exploring flavour-producing core microbiota in multispecies solid-state fermentation of traditional Chinese vinegar

**DOI:** 10.1038/srep26818

**Published:** 2016-05-31

**Authors:** Zong-Min Wang, Zhen-Ming Lu, Jin-Song Shi, Zheng-Hong Xu

**Affiliations:** 1 School of Pharmaceutical Science, Key Laboratory of Industrial Biotechnology of Ministry of Education, Jiangnan University, Wuxi 214122, China; 2 Tianjin Key Laboratory for Industrial Biological Systems and Bioprocessing Engineering, Tianjin Institute of Industrial Biotechnology, Chinese Academy of Sciences, Tianjin 300308, China; 3 National Engineering Research Centre of Solid-State Brewing, Luzhou 646000, China

## Abstract

Multispecies solid-state fermentation (MSSF), a natural fermentation process driven by reproducible microbiota, is an important technique to produce traditional fermented foods. Flavours, skeleton of fermented foods, was mostly produced by microbiota in food ecosystem. However, the association between microbiota and flavours and flavour-producing core microbiota are still poorly understood. Here, acetic acid fermentation (AAF) of Zhenjiang aromatic vinegar was taken as a typical case of MSSF. The structural and functional dynamics of microbiota during AAF process was determined by metagenomics and favour analyses. The dominant bacteria and fungi were identified as *Acetobacter*, *Lactobacillus*, *Aspergillus*, and *Alternaria*, respectively. Total 88 flavours including 2 sugars, 9 organic acids, 18 amino acids, and 59 volatile flavours were detected during AAF process. O2PLS-based correlation analysis between microbiota succession and flavours dynamics showed bacteria made more contribution to flavour formation than fungi. Seven genera including *Acetobacter*, *Lactobacillus*, *Enhydrobacter*, *Lactococcus*, *Gluconacetobacer*, *Bacillus* and *Staphylococcus* were determined as functional core microbiota for production of flavours in Zhenjiang aromatic vinegar, based on their dominance and functionality in microbial community. This study provides a perspective for bridging the gap between the phenotype and genotype of ecological system, and advances our understanding of MSSF mechanisms in Zhenjiang aromatic vinegar.

Multispecies solid-state fermentation (MSSF), is defined as a fermentation process in which multiple microorganisms grow on solid-state materials without present of free liquid. It might be one of the oldest and most economical ways of producing and preserving foods. It has been proved MSSF may improve the nutritional value, taste, smell, and healthy function of raw materials^1^,^2^. This traditional fermentation method is maintained through a spontaneous mixed-culture refreshment process without sterilisation. Enhanced by repeated practices for years, specific microbiota have been well characterised and their potential in food industry has been exploited intentionally^3^,^4^,^5^. It can be concluded the success of MSSF could rely on the reproducible formation of well-balanced microbiota, which determines the safety, smell, taste, texture, and aroma of fermented foods.

With the development of ecological techniques there are increasing studies to investigate food fermentation, focusing on the patterns/dynamics of the multi-species microbiota^4^,^5^,^6^,^7^ and the functionality of the microbial community^6^,^8^,^9^,^10^. These studies provide crucial information to help understand the role of microbiota and the function of the community in fermented foods. However, due to the complexity of MSSF and the lack of data mining strategy, the correlation between microbiota and flavours is still not clear^11^. Moreover, how to pick indicative functional core microbes from high species community, taking into account both dominance and functionality, is still challenging. Along with the advance of next generation sequencing, the principal research burdens are transforming from traditional wet-lab experiments to dealing with huge and informative data^12^. Bidirectional orthogonal partial least squares (O2PLS) method is an efficient statistic approach to integrate data collected from different analytical platform and dig into the potential associations between two disparate datasets^13^. This approach has been applied to investigate the metabolomic and proteomic correlation from mice samples^14^, the microbes and metabolic phenotype correlation in human gut^15^, and integrate transcript and metabolite data in plant biology^16^. However, there were scarce studies to inquire into associations between different omics platforms in fermented foods.

Zhenjiang aromatic vinegar, a well-known traditional fermented vinegar, is produced by three major steps including alcohol fermentation, acetic acid fermentation (AAF) and aging. Hereinto, AAF is a typical MSSF process with alcohol mash, wheat bran, and chaff as raw materials and fermented cereals from the last batch of AAF (termed *Pei* in Chinese, inoculum size, 8%, w/w) as starter^17^ (Fig. S1b). The succession of microbiota in the *Pei* during AAF process results in a dynamic flavours composition, which directly affects the taste and aroma of vinegars. Variation of flavours in fermented vinegar has been extensively studied by nuclear magnetic resonance spectroscopy, raman spectroscopy and mass spectrometry^18^,^19^,^20^,^21^,^22^,^23^. The microbial ecology during AAF process has also been investigated by culture-based and culture-independent approaches^4^,^5^,^6^,^7^. However, the correlation between microbiota and flavours and flavour-producing core microbiota remain to be determined in fermented vinegars.

To address this challenge, the assembly and dynamics of microbiota in vinegar *Pei* during AAF process were characterised by MiSeq sequencing. The changes of flavours composition during AAF were detected by chromatography and analysed by multivariable statistics. Based on these information, the relationship between microbiota assembly and flavours datasets was investigated by O2PLS. Finally, a functional core microbiota was selected by comparison of the comprehensive importance of microbiota correlated with flavours during AAF process.

## Results

### Phylogenetic landscapes and dynamics of microbiota during AAF process

**Figure 1:**
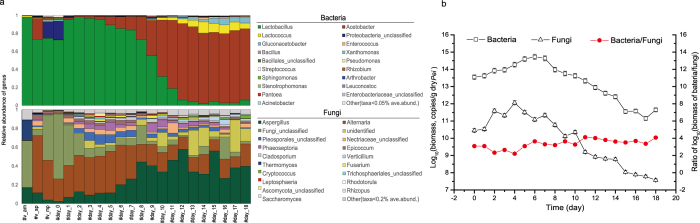
Distribution of microbiota in vinegar *Pei* and biomass of the bacteria and fungi in different samples during AAF process. (**a**) Average distribution of bacterial and fungal genera in vinegar *Pei* during AAF process. (**b**) Average biomass analysis of bacteria and fungi in vinegar *Pei* during AAF process.

PCR-based amplicon sequencing was applied to characterise the microbiota assembly and dynamics in vinegar *Pei* during AAF process. Across all samples, total 253 and 657 operational taxonomic units (OTUs) were detected for bacteria and fungi respectively with 97% similarity. The average of *Good’s coverage* was over 0.99 for all samples (Dataset S1), indicating the identified sequences represented majority of microbiota in vinegar *Pei*. Bacterial assembly were dominated by *Firmicutes* and *Proteobacteria*, while the fungi predominantly consisted of the phyla *Ascomycota*, *Fungi_unclassified*, and *Basidiomycota* (Fig. S2). A total of 151 bacterial genera and 202 fungal genera were identified in vinegar *Pei* during AAF process. As for bacteria, *Lactobacillus* was predominant in the early stage of AAF (days 0–9), while *Acetobacter*, *Lactococcus*, *Gluconacetobacter*, *Enterococcus*, and *Bacillus* were prevailing in the later stage of AAF (days 10–18). Therein *Acetobacter* could mainly originate from the starter cultures (#v_sp in Fig. 1a), and *Lactococcus* could mainly originate from alcohol mash (#v_am). *Gluconacetobacter*, *Enterococcus*, and *Bacillus* might originate from the raw materials (#v_mp), which were increasing with the proceeding of AAF (Fig. 1a). As for fungi, *Aspergillus* was existed in the whole AAF process, which increased in the early 13 days of AAF and then maintained fluctuation in small range (0.4–0.5). *Alternaria* was dominated in early stage of AAF (days 1–6), and then decreased gradually to the end of AAF. *Fungi_unclassified* accounted for more than 60% in sample #day_0 but declined rapidly once AAF started, which might originate from the alcohol mash (#v_am) and raw materials (#v_mp) (Fig. 1a). A total of 21 yeast genera were identified in vinegar *Pei*, including *Cryptococcus*, *Debaryomyces*, *Candida*, *Saccharomyces*, and so on (Fig. S3). However, these genera only accounted for 1.6% in fungal community. The biomass of bacteria was increasing in the first 7 days, and then decreased till the end of AAF while the biomass of fungi increased in the first 4 days, and then decreased till the end of AAF (Fig. 1b). Moreover, the biomass ratio of bacteria and fungi was in the range of 165 to 13,300, which suggested the bacteria played key role in the solid-state AAF.

### Comparison of the microbiota structure in vinegar *Pei* between different AAF stages

**Figure 2:**
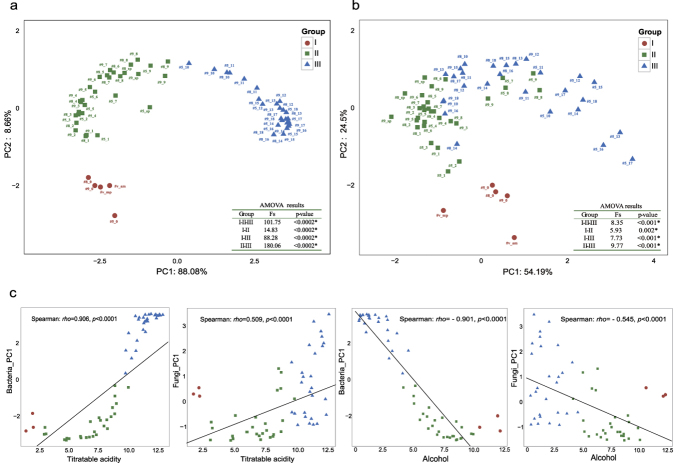
Comparison of the structure of microbiota in different samples and correlation between microbiota and environmental factors during AAF process. (**a**) PCA and AMOVA results of bacterial community in vinegar *Pei* at different stages of AAF based on *hellinger* distance with 97% similarity. (**b**) PCA and AMOVA results of fungal community in vinegar *Pei* at different stages of AAF based on *hellinger* distance with 97% similarity. (**c**) Correlation between the first principal component (PC1, bacteria and fungi) and titratable acidity and alcohol level respectively.

Though the dominant genera such as *Acetobacter*, *Lactobacillus* and *Aspergillus* were widely distributed across the vinegar *Pei*, their abundance within each sample is variable. Principal component analysis (PCA) was applied to compare the microbiota of vinegar *Pei* in different stages of AAF. It was shown that both bacterial and fungal community structure of the samples on day 0 of AAF exhibited little similarity to other samples except for raw material samples (#v_am, and #v_mp) (Fig. 2a,b). The samples in early stage were clustered separately from the samples in late stage of AAF based on the assembly and variation of microbiota, which indicated AAF process could be divided into three stages: I, day 0 (red circle in Fig. 2); II, days 1–9 (green box in Fig. 2); and III, days10–18 (blue triangle in Fig. 2). Furthermore, AMOVA showed that the degree of variation (*Fs*) among all stages was larger than within stages and *p*-value between any two stages of AAF (I vs. II, I vs. III, and II vs. III) was less than 0.001, suggesting the comparison of the divided three stages during AAF process was statistically significant (Fig. 2a,b). As for bacterial community, metastats analysis revealed a total of 38 OTUs, 21 OTUs, and 52 OTUs in stage I, II and III of AAF were significantly different from other two stages (*p* < 0.05) respectively. Therein, *Pseudomonas*, *Methylobacterium*, *Lactobacillus*, *Sphingomonas*, *Rhizobium*, *Staphylococcus*, *Xanthomonas* and *Acetobacter* were significant different genera in three stages. As for fungal community, it was shown that total 40 OTUs, 29 OTUs and 25 OTUs in stage I, II and III of AAF were significantly different from other two stages (*p* < 0.05) respectively, where *Aspergillus*, *Verticillium*, *Rhizomucor*, *Fungi_unclassified*, *Pleosporales_unclassified*, and *Eurotiales_unclassified* were significant different genera in three groups. Details of the bacterial and fungal taxonomy classification of the significant OTUs are listed in Tables S1, S2 and Dataset S1. In addition, the acidic stress and alcohol stress were two best predictors of bacterial and fungal community composition, with the principal coordinate one (PC1) being significantly associated with the gradient of titratable acidity and alcohol during AAF process (Fig. 2c, Bacteria: titratable acidity (*rho*, 0.906), alcohol (*rho*, −0.901); Fungi: titratable acidity (*rho*, 0.509), alcohol (*rho*, −0.545)), but the gradient of temperature was nearly not correlated with the bacterial and fungal community composition (Fig. S4, Bacteria: *rho*, −0.0974; Fungi: *rho*, −0.0859).

### Multivariate analysis of flavours during AAF process

**Figure 3:**
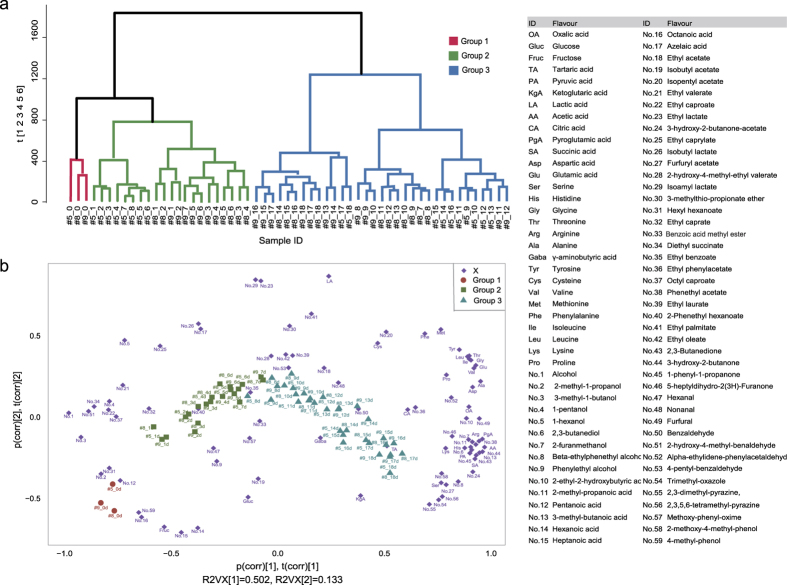
PCA and HCA analysis of flavours in vinegar *Pei* during AAF process. (**a**) The dendrogram of AAF process was obtained by hierarchical cluster analysis based on PCA modeling. (**b**) The biplot superimposed the scores and loadings of PCA analysis based on correlation scaling method. *R2VX* represents the fraction of *X* variation modeled in the component. *p*(*corr*), *t*(*corr*) is a combined vector, *p*(*corr*) represents loading *p* scaled as correlation coefficient between *X* and *t*; *t*(*corr*) represents score *t* scaled as correlation coefficient resulting in all points falling inside the circle with radius 1.

A total of 88 flavours were detected during AAF process, including 2 sugars, 9 organic acids (OAs), 18 amino acids (AAs) and 59 volatile flavours (VFs). The volatile flavours could be divided into seven categories including 9 alcohols (No. 1–9), 8 acids (No. 10–17), 25 esters (No. 18–42), 4 ketones (No. 43–46), 7 aldehydes (No. 47–53), 3 heterocycles (No. 54–56), and 3 others (No. 57–59) (Fig. S5). PCA analysis showed that the first two components *R*^*2*^*X*(*cum*) explained 63.2% of the variables and the cross-validated *Q*^*2*^-value for each component were more than the cross validation threshold for that component (*Limit*), indicating significant components for this analysis (Dataset S2). The projected coordinate of metabolites in PC1 appeared to capture the evolutionary tendency of flavours during AAF process, and dynamics of flavours were clearly distinct in different stages of AAF (Fig. 3b). Hierarchical cluster analysis (HCA) revealed the AAF process could be divided into 3 groups based on flavours: group1, day 0; group 2, days 1–7; group 3, days 8–18 (labelled red, green, and blue in Fig. 3a respectively). A biplot integrating scores and loadings demonstrated there were 13 flavours including fructose, glucose, and 11 VFs highly correlated with group 1 (red circle in Fig. 3b); 15 VFs highly correlated with group 2 (green box in Fig. 3b); and 60 flavours including 9 OAs, 18 AAs and 33 VFs highly correlated with group 3 (blue triangle in Fig. 3b). More detailed information is provided in Table S3. These results suggested most of the flavours (OAs, AAs and half of VFs) were produced in the late stages of AAF (days 8–18).

### Association between microbiota and flavours during AAF process

**Figure 4:**
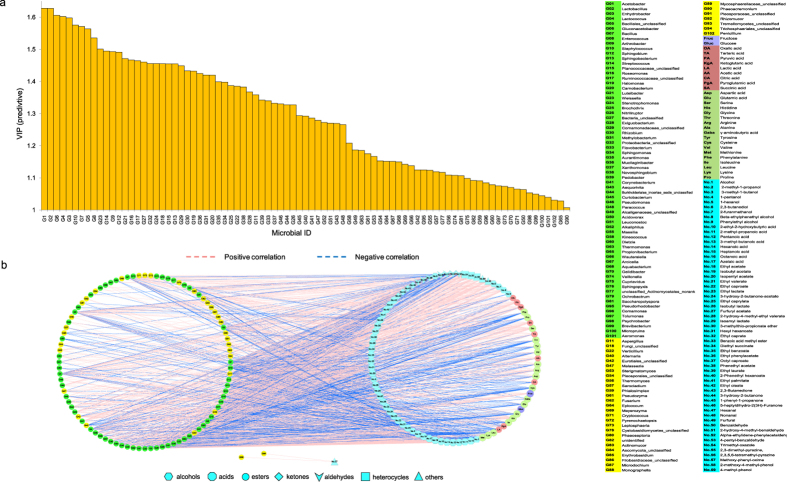
Correlation analyses between microbiota and flavours by O2PLS modeling during AAF process. (**a**) *VIP*_(*pred*)_ (variable importance for predictive components) plot of the important microbiota (*VIP*_(*pred*)_ > 1.0). (**b**) The correlated network between microbial genera and flavours during AAF process. The left-side circles represent the bacterial (green) and fungal (yellow) genera correlated with flavours (|*ρ*| > 0.7). The right-side circles represent the flavours (sugars, light purple circle; organic acids, light red circle; amino acids, light green circle; volatile flavours, light blue labels (hexagons: alcohols; octagons: acids; circles: esters; diamonds: ketones; vees: aldehydes; rects: heterocycles; triangles: others)) correlated with microbiota (|*ρ*| > 0.7). The red long dashed lines linking the circles represent positive correlation while the blue long dashed lines represent the negative correlation between microbiota and flavours.

O2PLS method was used to analyse the association between microbiota and flavours during AAF process. It was shown *R*^*2*^ and *Q*^*2*^ of the model was 0.879 and 0.528 respectively (Dataset S2, Fig. S6a), suggesting O2PLS method was well fitted for analysis and prediction. The first two predictive components were significant by cross validation, accounting for 90% of *R*^*2*^(*cum*) and 100% of *Q*^*2*^(*cum*) in this model (Fig. S6b). The *VIP*_(*pred*)_ vector (*VIP* value for the predictive components) of analysed microbiota varied in 0.15–1.63, in which total 85 microbial genera (*VIP*_(*pred*)_ > 1.0) including 66 bacterial genera (*VIP*_(*pred*)_ ≈ 1.03–1.63) and 19 fungal genera (*VIP*_(*pred*)_ ≈ 1.01–1.46) had important effects on the flavours (Fig. 4a, Dataset S2), suggesting bacteria were more important for vinegar production than fungi. *Acetobacter*, *Lactobacillus*, *Gluconacetobacter*, and *Lactococcus* were the biggest contributors to the production of flavours during AAF process. Based on correlation coefficient between microbiota and flavours, a total of 94 genera including 61 bacteria (green circles in left side of Fig. 4b) and 33 fungi (yellow circles in left side of Fig. 4b) were moderately and highly correlated (|*ρ*| > 0.7) with all three flavour sets, in which total 47 genera (36 bacteria and 11 fungi) were correlated with OAs (light red circles in right side of Fig. 4b); 59 genera (48 bacteria and 11 fungi) were correlated with AAs (light green circles in right side of Fig. 4b); and 92 genera (61 bacteria and 31 fungi) were correlated with VFs (light blue labels in right side of Fig. 4b). *Acetobacter* and *Lactobacillus* possessed the largest number of correlated flavours (56 and 53 respectively), while *Aspergillus* and *Fungi_unclassified* were correlated with 39 and 34 of flavours respectively (|*ρ*| > 0.7) (Table S8). Most of fungal genera (75.7%) had correlated with few flavours (≤5), in which 14 genera had poor correlated with only one flavour. Details of the relationships between the microbiota and flavours are listed in Table S4 and Table S8.

For OAs, bacteria played more important role than fungi, in which *Lactobacillus*, *Enhydrobacter*, and *Gluconacetobacter* were important genera for the production of OAs during AAF process. Acetic acid (AA) and lactic acid (LA) were main acids in cereal vinegar. Total 25 genera were correlated with AA (|*ρ*| > 0.7) (Fig. 4b, Table S5), in which *Acetobacter*, *Enhydrobacter*, and *Lactobacillus* had excellent correlation with AA (|*ρ*| > 0.9), indicating the three genera were mainly responsible for the change of AA during AAF process. LA was positively correlated with *Phaeoseptoria* and *Fusarium*; and negatively correlated with 14 genera during AAF process. Therein *Staphylococcus* and *Weissella* were two most important genera for change of LA during AAF process. Detailed information of genera correlated with each organic acid is summarised in Table S5.

For AAs, *Acetobacter*, *Aspergillus*, *Lactobacillus*, *Enhydrobacter*, *Roseomonas*, *Sphingobacterium*, *Staphylococcus*, *Stenotrophomonas*, and *Fungi_unclassified* were crucial to dynamics of AAs during AAF process (Fig. 4b). Glutamic acid (Glu), alanine (Ala), valine (Val), and leucine (Leu) are four abundant flavours for the taste of vinegar. Glu and Leu, providing umami and bitter taste of vinegar, were correlated with 14 and 22 genera (|*ρ*| > 0.7) respectively, in which *Staphylococcus*, *Acetobacter*, *Sphingobacterium*, and *Aspergillus* were highly correlated with the changes of Glu and Leu during AAF process (|*ρ*| > 0.8). Ala, providing sweet taste of vinegar, was correlated with 16 genera (|*ρ*| > 0.7), in which *Acetobacter*, *Aspergillus*, *Staphylococcus*, and *Lactobacillus* were most important (|*ρ*| > 0.8) for the change of Ala during AAF process. Val, providing sweet and bitter taste of vinegar, was correlated with 14 genera (|*ρ*| > 0.7), in which *Acetobacter*, *Aspergillus*, *Sphingobacterium*, and *Staphylococcus* were the major Val producers (|*ρ*| > 0.8). Moreover, γ-aminobutyric acid (Gaba), a bioactive component in vinegar, has physiological functions to depress the elevation of systolic blood pressure^24^. Change of Gaba during AAF process was correlated with 7 genera (|*ρ*| > 0.6), in which *Epicoccum* and *Alternaria* were the most important genera. Details of correlated genera with each amino acid are listed in Table S6.

*Acetobacter*, *Lactococcus*, *Lactobacillus*, and *Gluconacetobacer* were important to dynamics of VFs during AAF process, which were correlated with more than 30 VFs (|*ρ*| > 0.7) (Fig. 4b, Table S8). A total of 56 genera were correlated with 9 volatile alcohols (|*ρ*| > 0.7), in which *Acetobacter*, *Lactobacillus*, *Enhydrobacter*, *Lactococcus*, *Bacillales_unclassified*, *Gluconacetobacer*, *Enterococcus*, *Arthrobacter*, *Carnobacterium*, *Verticillium*, and *Nitriliruptor* were correlated with more than 7 alcohols (light blue hexagons in Fig. 4b). Total 51 genera were correlated with 8 volatile acids and most of the correlation were positive (|*ρ*| > 0.7) (light blue octagons in Fig. 4b). There were 81 genera correlated with 25 volatile esters (|*ρ*| > 0.7), in which most of fungal genera were correlated with few esters (≤4) (light blue circles in Fig. 4b). There were 27 genera correlated with 4 volatile ketones (|*ρ*| > 0.7) and most of the correlation were positive (light blue diamonds in Fig. 4b). There were 48 genera correlated with 7 volatile aldehydes (|*ρ*| > 0.7), in which *Staphylococcus* and *Sphingobacterium* were correlated with more than 5 aldehydes (light blue vees in Fig. 4b). Total 21 genera were correlated with 3 volatile heterocycles, and the correlation are positive except *Lactobacillus* (light blue rects in Fig. 4b). 2,3,5,6-tetramethyl-pyrazine (No. 56, known as ligustrazine), a functional bioactivator in vinegar, was correlated with 16 genera, in which *Gluconacetobacer*, *Ruminococcaceae_unclassified*, and *Sphingobium* were excellently correlated with change of ligustrazine (|*ρ*| > 0.9). Total 50 genera were correlated with 3 others volatile (|*ρ*| > 0.7)(light blue triangles in Fig. 4b). In addition, there were 9 volatile flavours exhibited a weak correlation with microbiota (|*ρ*| < 0.7), suggesting these metabolites might be produced by natural physiochemical process. Details of the microbiota correlated with each volatile flavour are listed in Table S7.

### Analysis of the functional core microbiota for vinegar fermentation

**Figure 5:**
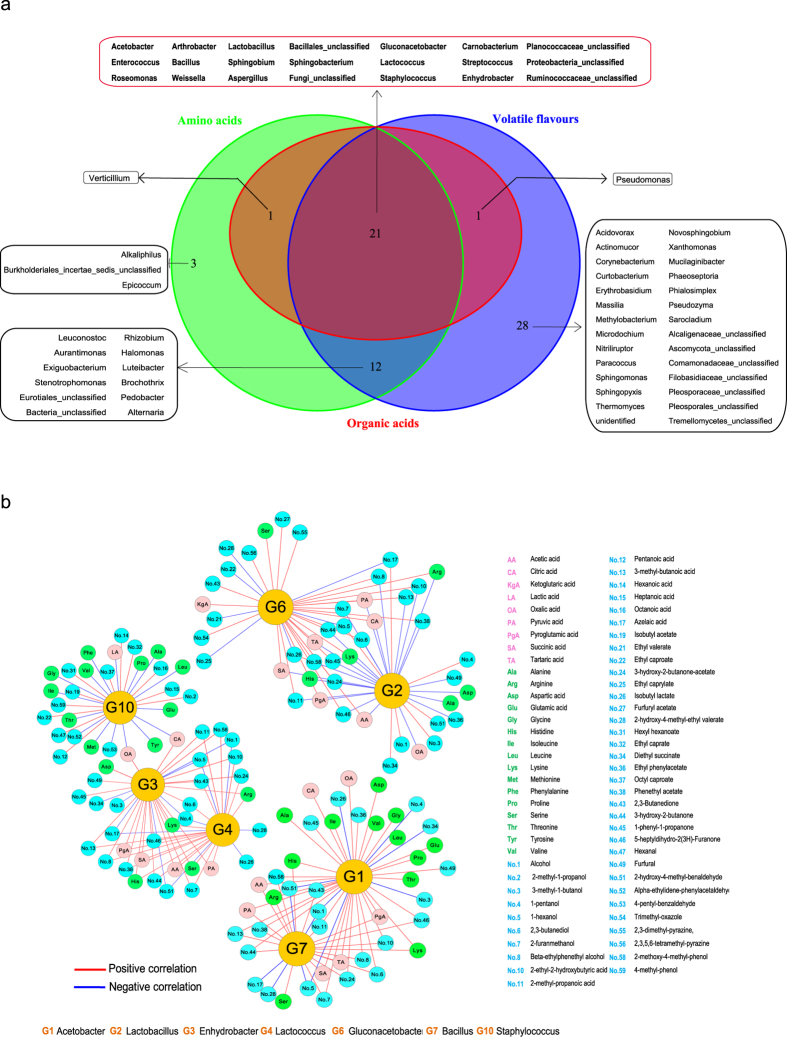
Analysis of the core microbiota for vinegar *Pei* during AAF process. (**a**) Venn diagram of relationship of microbiota highly correlated with organic acids, amino acids, and volatile flavours (|*ρ*| > 0.8). (**b**) The core microbiota accord with the following terms: (i) detected stably in the whole process of AAF; (ii) the shared microbiota for three flavour sets; (iii) the *VIP*_(*pred*)_ value of microbiota was greater than 1.55; (iv) the number of flavours highly correlated with microbiota (|*ρ*| > 0.8) was greater than 25.

Further analysis was performed to investigate the relationship of microbiota highly correlated with three flavour sets in vinegar *Pei* during AAF process (|*ρ*| > 0.8) (Fig. 5a, Table S9). It was shown there were 23, 37 and 62 genera highly correlated with OAs, AAs and VFs respectively. Total 21 genera including 19 bacterial genera and 2 fungal genera were common to three flavour sets. Fungal genera highly correlated with VFs were more than OAs and AAs, which indicated the fungal community in vinegar *Pei* were partly contributed to the aroma and fragrance of vinegar. In order to study the functional core microbiota in vinegar *Pei*, several conditions should be considered: (i) detected stably in AAF process; (ii) the shared microbiota among three flavour sets; (iii) the *VIP*_(*pred*)_ value of microbe was greater than 1.55; (iv) the number of flavours highly correlated with microbiota (|*ρ*| > 0.8) was greater than 25. Based on these, seven genera including *Acetobacter* (G1), *Lactobacillus* (G2), *Enhydrobacter* (G3), *Lactococcus* (G4), *Gluconacetobacer* (G6), *Bacillus* (G7) and *Staphylococcus* (G10) were selected as functional core microbiota for AAF of Zhenjiang aromatic vinegar (Fig. 5b). These seven genera were highly correlated with dynamics of 69 flavours during AAF process, including 9 OAs, 16 AAs, and 44 VFs (|*ρ*| > 0.8). Therein, *Acetobacter*, *Gluconacetobacer*, *Lactobacillus*, and *Enhydrobacter* were mainly responsible for the change of OAs, while *Acetobacter* and *Staphylococcus* were mainly responsible for the change of AAs. These 7 genera were all contributed to the change of VFs, in which *Acetobacter*, *Lactobacillus*, and *Enhydrobacter* were mainly responsible for that of volatile alcohols; *Gluconacetobacer* was mainly responsible for changes of volatile esters and heterocycles; and *Staphylococcus* was mainly responsible for volatile aldehydes. More detailed information about the functional core microbiota is listed in Table S10. In addition, PICRUSt analysis revealed that the predicted functions of the core microbiota and non-core microbiota were all assigned to seven categories including metabolism, unclassified, genetic information processing, environmental information processing, organismal systems, cellular processes, and none (Fig. S7). Therein, metabolism was the main function (39.11%) of the microbial community in vinegar *Pei,* mainly including amino acid metabolism, carbohydrate metabolism, and biosynthesis of other secondary metabolites (Dataset S2). The core microbiota could contribute to 87.87% of metabolism function (Fig. S7). Genetic information processing and environmental information processing were essential to the microbial community in vinegar *Pei* (occupied 21.49% and 13.33% respectively), which were also mainly carried out (88.60%) by core microbiota (Fig. S7). These suggested the core microbiota could perform the most function of the total microbial community in vinegar production.

## Discussion

Microbiota inhabiting in vinegar *Pei* is of great importance for the quality and characteristics of cereal vinegars. Many molecular ecological approaches have been used to characterise the bacterial and fungal community^4^,^5^,^25^,^26^,^27^. In this study, 151 bacterial genera and 202 fungal genera in vinegar *Pei* during AAF process were identified by next generation sequencing, revealing higher diversity and quantitative abundance than previous studies^4^,^5^,^28^,^29^,^30^,^31^,^32^. The majority of sequences in vinegar *Pei* were assigned to *Acetobacter*, *Lactobacillus*, *Aspergillus*, and *Alternaria*, which were consisted with the previous studies^4^,^26^. *Acetobacter* was increased during AAF process while *Lactobacillus* was gradually decreased. This succession tendency might be as a potential indicator to ensure the normal AAF process. Yeast community in vinegar *Pei* included 21 identified genera in this study, suggesting higher diversity than that in Tianjin duliu mature vinegar^26^ and traditional balsamic vinegar^33^. However, the abundance of yeast was only occupied 1.6% in fungal genera, and the conjecture was yeast autolysis occurred after alcohol fermentation^34^,^35^. During manufacturing, the AAF process is controlled empirically, and the complexity of microbiota make it difficult to be used as a rational approach to monitor AAF process. Here, Miseq sequencing provided well depth to cover the complex microbiota in vinegar *Pei*. Based on structure of microbiota, the AAF process was divided into three distinct stages: I, day 0; II, days 1–9; and III, days 10–18. This division provided a succession profile of microbiota during AAF, which could be used to search for microbial markers characterising the AAF process and develop a microbiota-based strategy to monitor AAF process. Moreover, the correlation between the microbial succession and environmental factors showed that the gradient of titratable acidity was the most important driver to promote succession of microbiota (Fig. 2c). Elevated levels of AA and LA in vinegar *Pei* resulted in a specially acidic stress, which selected most of acid-tolerant microbes such as *Ga. europaeus*^29^. Another important factor was the alcohol stress, which is a preferred carbon source for growth of functional microbes during vinegar production^36^. Eventually, a well-balanced and robust community is formed via long-time environmental selection.

It is interesting that the grouping of AAF process based on microbial assembly (day 0, days 1–9, and days 10–18) is basically accordance with the grouping based on flavours (day 0, days 1–7, and days 8–18) (Figs 2 and 3b), suggesting the uniformity and high correlation between the evolution of microbiota and the change of flavours. There are few investigations of the correlation between microbiota and flavours in traditional fermented foods^6^. Here, O2PLS approach was used to integrate the microbiota dataset and flavours dataset in order to dig into the association between microbiota and flavours in vinegar *Pei* during MSSF process. In this study, more bacterial genera showed a higher correlation with three flavour sets (|*ρ*| > 0.8) than fungal genera (Fig. 4), which indicated bacterial community might be the main producer for vinegar flavours. Seven genera including *Acetobacter*, *Lactobacillus*, *Enhydrobacter*, *Lactococcus*, *Gluconacetobacer*, *Bacillus*, and *Staphylococcus* were selected as functional core microbiota for AAF of Zhenjiang aromatic vinegar. Moreover, the function of core microbiota predicted by PICRUSt analysis accounted for more than 80% of functions of the microbial community in vinegar *Pei*. Among seven functional genera, *Acetobacter* and *Lactobacillus* are major functional microbes that have been studied extensively in vinegar industry^4^,^29^. In the further work, *A. pasteurianus*, a main species isolated from vinegar *Pei*, was added at the beginning of AAF to augment the flavours production of Zhenjiang aromatic vinegar. The result showed that the temperature of vinegar *Pei* in AAF process augmented with *A. pasteurianus* increased faster than that in the non-augmented AAF process (control) (Fig. S8a). The level of total acids in the AAF process of adding *A. pasteurianus* was higher than control, and the level of total acids at the 15^th^ day of AAF process of adding *A. pasteurianus* was equivalent to the total acids at the 18^th^ day of control AAF process (Fig. S8b), which suggested the fermented period might be shortened by adding *A. pasteurianus*. Moreover, variety of flavours such as AA, Glu, 2,3-butanediol, and ligustrazine (Table S11) were increased at the end of augmented AAF process compared with the control, which partly validated the correlation between *Acetobacter* and flavours. The structure and dynamics of microbiota after adding *A. pasteurianus* are being studied. According to our knowledge, this is the first report to systemic analyse the relationship between the structure (genotype) and function (phenotype) of microbial community in traditional fermented foods.

## Methods

### Study design and sampling

The framework of the experiment design was shown in Fig. S1a. The AAF of Zhenjiang aromatic vinegar was carried out from July to October, 2014 in Jiangsu Hengshun Vinegar Industry Co., Ltd., China. Vinegar *Pei* from three randomly selected AAF batches (denoted as #5, #8 and #9) were sampled every day using a sterilized cylinder-shaped sampler (Puluody, Xi’an, China). Meanwhile, the alcohol mash (#v_am), starter *Pei* (#v_sp) and a mixture of raw materials (#v_mp) including alcohol mash, wheat bran, chaff and starter *Pei* were collected. In order to obtain the most unbiased samples, vinegar *Pei* at the four vertexes and the centre of the pool were collected from top to bottom, mixed thoroughly, and then reduced by coning and quartering repeatedly (Fig. S1c). About 500 g of sample was sealed in a sterile plastic bag, and stored at −20 °C before further analysis. During the AAF process, the temperature and moisture content of vinegar *Pei* were 34–46 °C and 60–70%, respectively. The AAF lasted 18 days, and a total of 62 fresh samples were obtained for further analysis. Detailed information of samples is shown in Dataset S1.

### DNA extraction, amplicon and sequencing

DNA extraction using the CTAB-based method was applied in this study^37^. For bacteria, the V4–V5 domains of 16S rRNA genes were amplified using primers 515F and 907R^38^. For fungi, the internal transcribed spacer (ITS) region were amplified with primers 1737F and 2043R^39^. The sequences of primers are listed in Table S12. Amplicons were submitted to the Majorbio Bio-Pharm Technology Co., Ltd. (Shanghai, China) for illumina paired-end library preparation, cluster generation, and 300-bp paired-end sequencing on a MiSeq instrument in two separate runs. The run of bacterial 16S rRNA generated 1,257,819 reads (396.42 nt mean length) and the run of fungal ITS generated 1,224,296 reads (263.41 nt mean length). Details of the DNA extraction and PCR amplification are described in Supplemental information.

### Microbial biomass analysis by quantitative real-time PCR

To estimate the biomass of bacteria and fungi during the AAF process of Zhenjiang aromatic vinegar, qRT-PCR was performed using a CFX connect Real-Time system (Bio-Rad, California, US) with commercial kit (SYBR Premix Ex Taq, Takara, Dalian, China). The total genomic DNA from *Pei* was measured (Nanodrop 2000, Wilmington, US) and used as the template to amplify bacteria using primers^40^ 340F and 758R and fungi using primers^5^ Y1 and Y2. The specificity of amplification was determined by melting curve analysis. For determination of the number of bacterial and fungal amount in each sample, fluorescent signals, detected from 10 times serial dilution (from 10E + 14 copies/μL to 10E + 3 copies/μL) in the linear range of the assay, were averaged and compared to a standard curve generated with standard DNA in the same experiment^41^. The sequences of primers are listed in Table S12. Details of PCR amplification are described in Supplemental information.

### Sequence processing and community structure analysis

Raw reads were de-multiplexed, quality-filtered, and analysed using QIIME (v.1.17)^42^. The representative OTU sequences were annotated using the RDP bacterial 16S rRNA database (Release 11.1) and the UNITE fungal ITS database (Release 6.0)^43^ by a QIIME-based wrapper of RDP-classifier (v.2.2)^44^. Alpha-diversity and β-diversity estimates were calculated using *hellinger* distance between samples for bacterial 16S rRNA reads and fungal ITS reads with 97% identity. Principal component were computed from the resulting distance matrices to compress dimensionality and visualise the relationships between samples according to PCA plots^45^. To determine whether sample classifications (different fermentation phase) contained differences in phylogenetic or species diversity, analysis of molecular variance (AMOVA)^46^ was used to test significant differences between sample groups based on *hellinger* distance matrices. Metastats was used to determine which taxa resulted in these differences between sample groups^47^. Moreover, environmental conditions do correlate with variation in community composition; spearman correlation was applied to explore the potential determiner for the succession of bacterial and fungal community (the first principal component) in vinegar *Pei*. Details of the sequence processing and statistical analyses are summarized in Supplemental information.

### Flavours analysis and multivariate data analysis

The contents of fructose, glucose, OAs, AAs, and VFs were detected by chromatography. PCA and HCA were used to investigate the flavours data during AAF process. In HCA, the distance between observations was calculated using *Ward’s* method. In PCA, we superimposed the score vectors and loading vectors based on the correlation scaling method, leading to the new vectors *t*(*corr*) and *p*(*corr*). Then, the new vectors of the first two components were visualized by a biplot. According to the relative positions between observations and variables, we were able to determine which flavours were highly correlated with each AAF group. Before analysis, the flavours data were normalised using the min-max method. PCA and HCA were performed in SIMCA 14 (demo v.1.0.1) (Umetrics AB, Umeå, Sweden). Details of flavours analysis are summarized in Supplemental information.

### Correlation analysis between microbiota and flavours during AAF process

As for microbiota in *Pei*, the top 100 bacterial genera and top 100 fungal genera were further analysed according to rank of sum of abundance. For flavours, total 88 flavours including 2 sugars, 9 OAs, 18 AAs and 59 VFs were applied to investigate the relationship with microbiota. O2PLS modelling was used to unveil the association between microbiota at genus level and each flavour during AAF, in which, microbiota data for 200 genera (defined as *X* matrix) were mapped to flavours data (defined as *Y* matrix)^16^. O2PLS method consists of simultaneous projection of both the *X* and *Y* matrices on low dimensional hyper planes^13^. The number of components in respective set of O2PLS model is evaluated by seven-fold cross-validation. Variable Importance in the Projection (*VIP*) and a pair-wise correlation matrix (|*ρ*| > 0.7) were employed to identify potential functional microbiota in vinegar *Pei*. Terms with larger *VIP* value (>1), are the most relevant for explaining *Y* variables. The correlation matrix shows the pair-wise correlation between all variables (*X* and *Y*), in which the value of correlation coefficient represents the extent of the linear association between the two terms, ranging from −1 to 1. O2PLS analysis was performed using the SIMCA 14 (demo v.1.0.1) (Umetrics AB, Umeå, Sweden). Further statistic analyses and graphics were performed in Microsoft® Excel and R software (v.2.14.1). The correlation between microbiota and flavours was visualised via Cytoscape (v.2.8.3). Details for correlation analysis are listed in Supplemental information.

### Predicted function of the core microbiota and non-core microbiota in vinegar *Pei*

To validate the function of the core microbiota for the whole community in vinegar *Pei*, phylogenetic investigation of communities by reconstruction of unobserved states (PICRUSt), was used to predict which gene families were present^48^. Given that the copy number and function of bacteria were more abundant than fungi, PICRUSt was performed base on the bacterial 16S gene surveys. For this analysis, OTUs were closed-reference picked against the Greengenes by QIIME (v.1.7). The functional core taxonomies were filtered as a separate dataset of core microbiota while the remaining taxonomies were regarded as another dataset of non-core microbiota. The two datasets were normalised, predicted, and categorised according to online protocols of PICRUSt (http://huttenhower.sph.harvard.edu/galaxy). The predicted functions of the core microbiota and non-core microbiota were compared and visualised in Microsoft^®^ Excel and Origin (v.8.0).

## Additional Information

**Accession codes:** The sequences data reported in this paper have been deposited in the GenBank database (No. SRP059163).

**How to cite this article**: Wang, Z.-M. *et al.* Exploring flavour-producing core microbiota in multispecies solid-state fermentation of traditional Chinese vinegar. *Sci. Rep.* **6**, 26818; doi: 10.1038/srep26818 (2016).

## Supplementary Material

Supplementary Information

Supplementary Dataset 1

Supplementary Dataset 2
