# Supplementary material for: Exploring flavour-producing core microbiota in multispecies solid-state fermentation of traditional Chinese vinegar
Source: Sci Rep. 2016 May 31;6:26818. doi: 10.1038/srep26818 (PMC4886211; doi:10.1038/srep26818)
Supplement: Supplementary Information [file srep26818-s1.doc]

**Supplemental Information for**

**Flavour-producing core microbiota in multispecies solid-state fermentation process of traditional Chinese vinegar**

Zong-Min Wang ^1,†^, Zhen-Ming Lu ^1,2,†^, Jin-Song Shi^1,3^ & Zheng-Hong Xu ^1,2,3^,*

*^1^School of Pharmaceutical Science, Key Laboratory of Industrial Biotechnology of Ministry of Education, Jiangnan University, Wuxi 214122, China;*

*^2^Tianjin Key Laboratory for Industrial Biological Systems and Bioprocessing Engineering, Tianjin Institute of Industrial Biotechnology, Chinese Academy of Sciences, Tianjin, 300308;*

*^3^ National Engineering Research Centre of Solid-State Brewing, Luzhou 646000, China;*

*Correspondence to: [zhenghxu@jiangnan.edu.cn](mailto:zhenghxu@jiangnan.edu.cn)

**This file includes:**

Supplementary figures (Figure S1-S8)

Supplementary tables (Table S1-S12)

Supplementary methods and references

**Other supplementary information for this manuscript includes the following:**

Supplementary dataset 1 as an Excel file: Supplementary_Dataset_S1.xls

Supplementary dataset 2 as an Excel file: Supplementary_Dataset_S2.xls

**Supplementary figure legends**

**Figure S1.** Experimental procedure **(a)**, technical process of Zhenjiang aromatic vinegar **(b)** and diagram of sampling location **(c)**.

**Figure S2.** Average distribution of bacterial and fungal phyla in vinegar *Pei* during AAF process of Zhenjiang aromatic vinegar.

**Figure S3.** Diversity of yeast community at genus level in vinegar *Pei* samples.

**Figure S4.** Correlation between the first principal components of **(a)** bacteria and **(b)** fungi and temperature.

**Figure S5.** Detected flavours in vinegar *Pei* during AAF process of Zhenjiang aromatic vinegar.

**Figure S6.** Summary of fit and overview plots for O2PLS model. **(a)** The summary fit plot displays the cumulative *R^2^* and *Q^2^* for the *Y*-matrix modeled by *X*. The labeled *P* in *x*-axis represents for predictive components. **(b)** The overview plot provides a graphical summary of the predictive and orthogonal sources of variation in the O2PLS model. The column is both stacked and color coded in accordance with the predictive and orthogonal variation structures.

**Figure S7.** Predicted functions of the core microbiota and non-core microbiota in vinegar *Pei* by PICRUSt.

**Figure S8.** Changes of temperature **(a)** and total acids **(b)** in AAF process bioaugmented with Acetobacter pasteurianus.

**Supplemental figures**

**
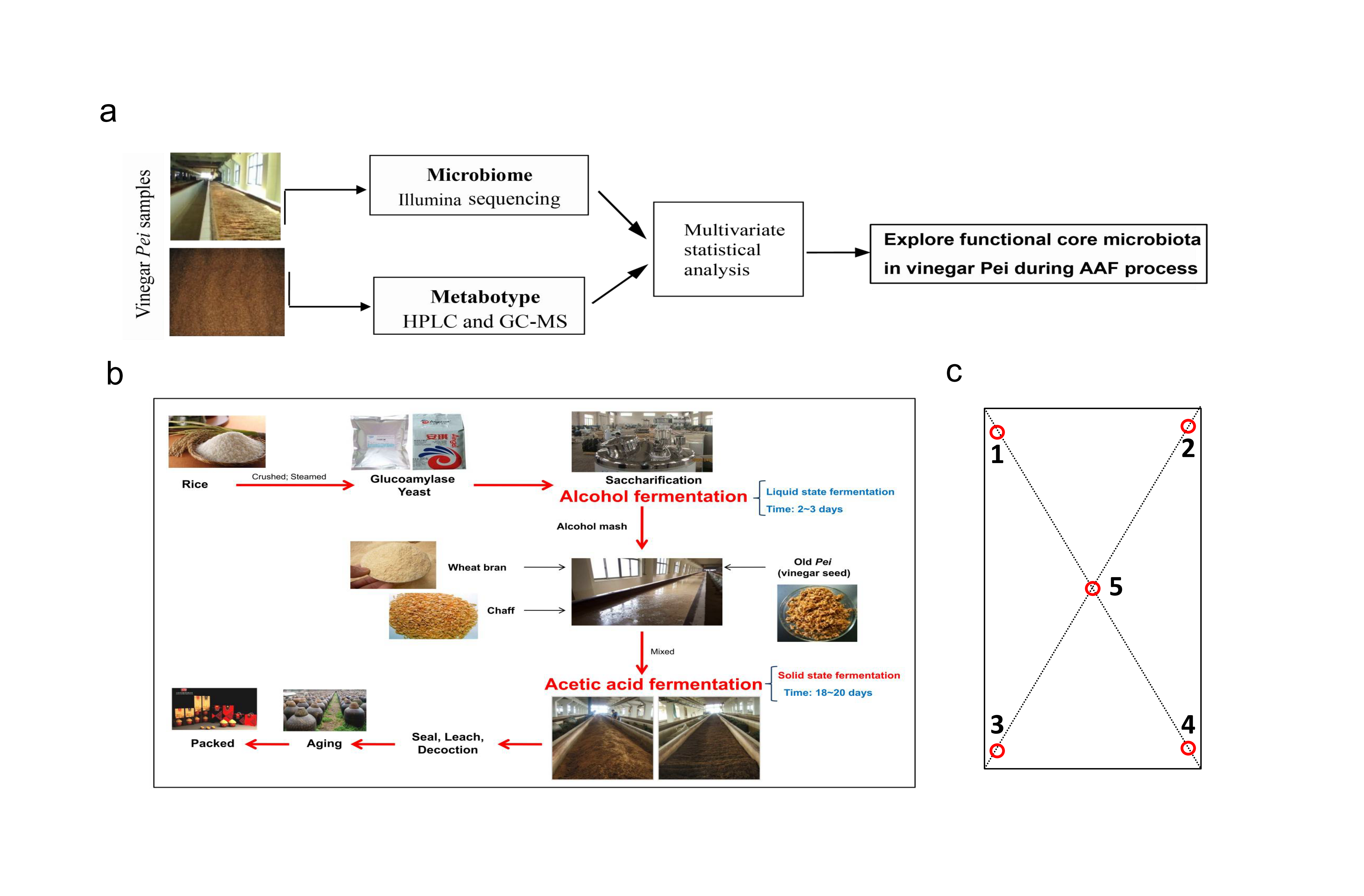
**

**Figure S1.** Experimental procedure **(a)**, technical process of Zhenjiang aromatic vinegar **(b)** and diagram of sampling location **(c)**.

**
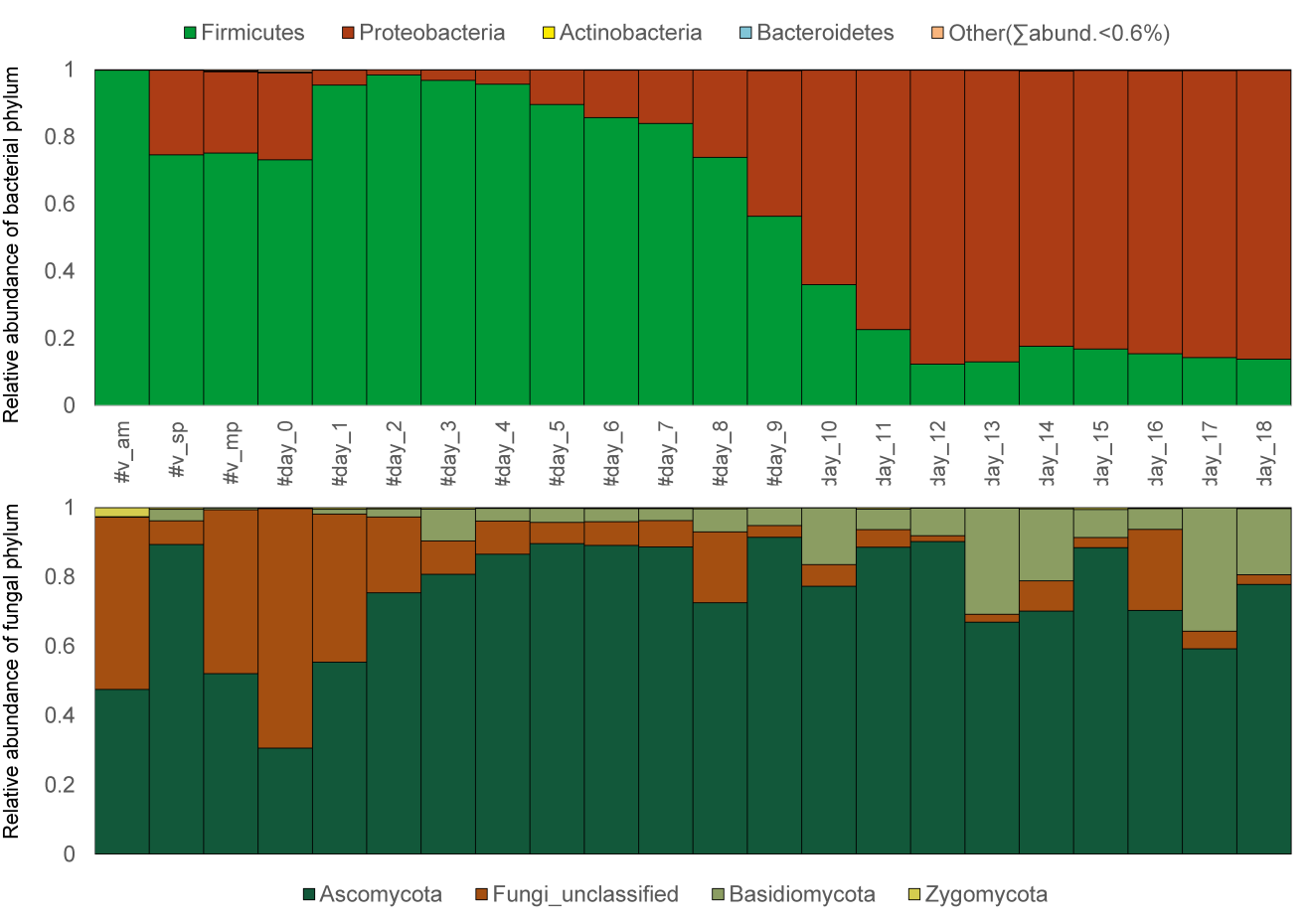
**

**Figure S2.** Average distribution of bacterial and fungal phyla in vinegar *Pei* during AAF process of Zhenjiang aromatic vinegar.

**
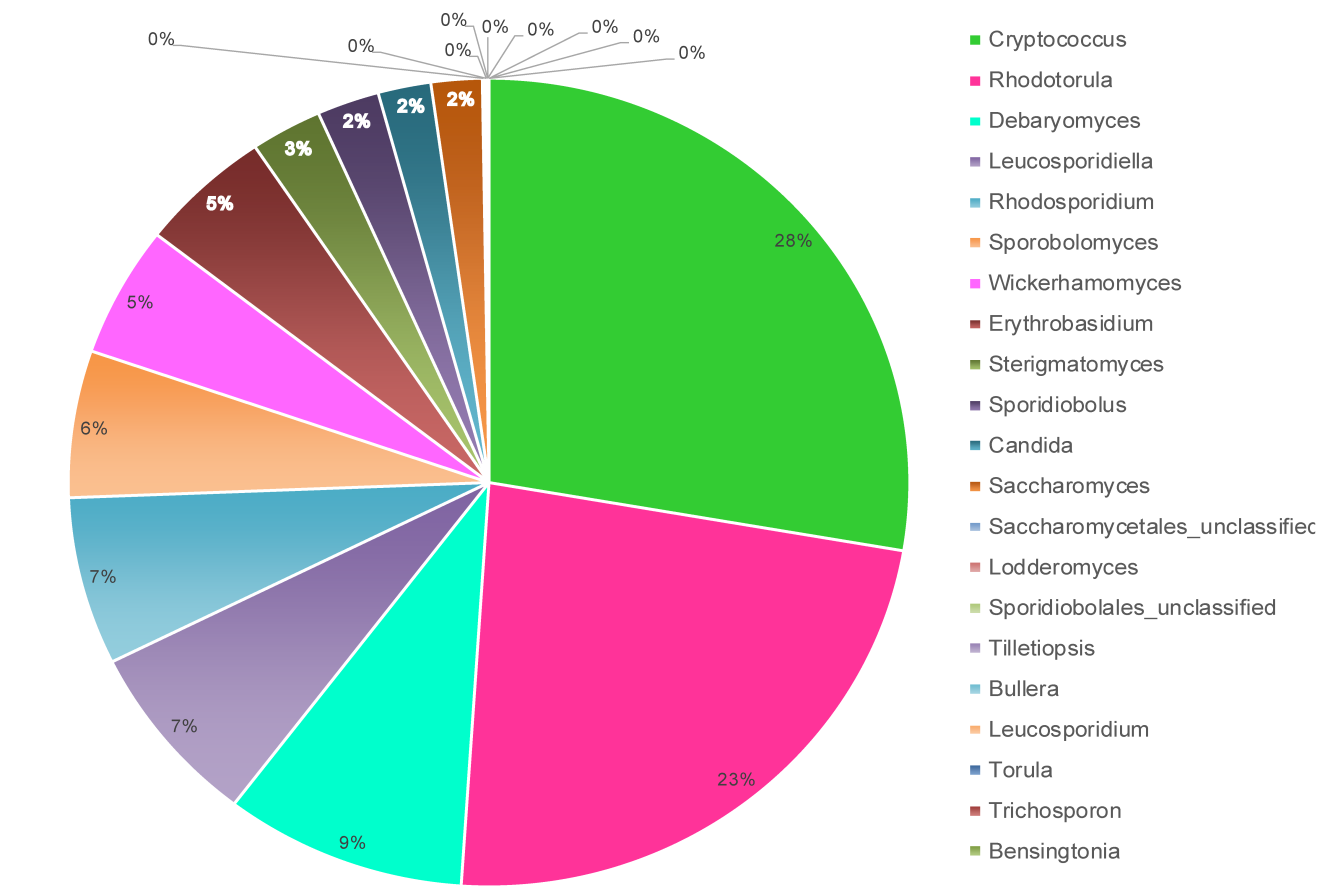
**

**Figure S3.** Diversity of yeast community at genus level in vinegar *Pei* samples


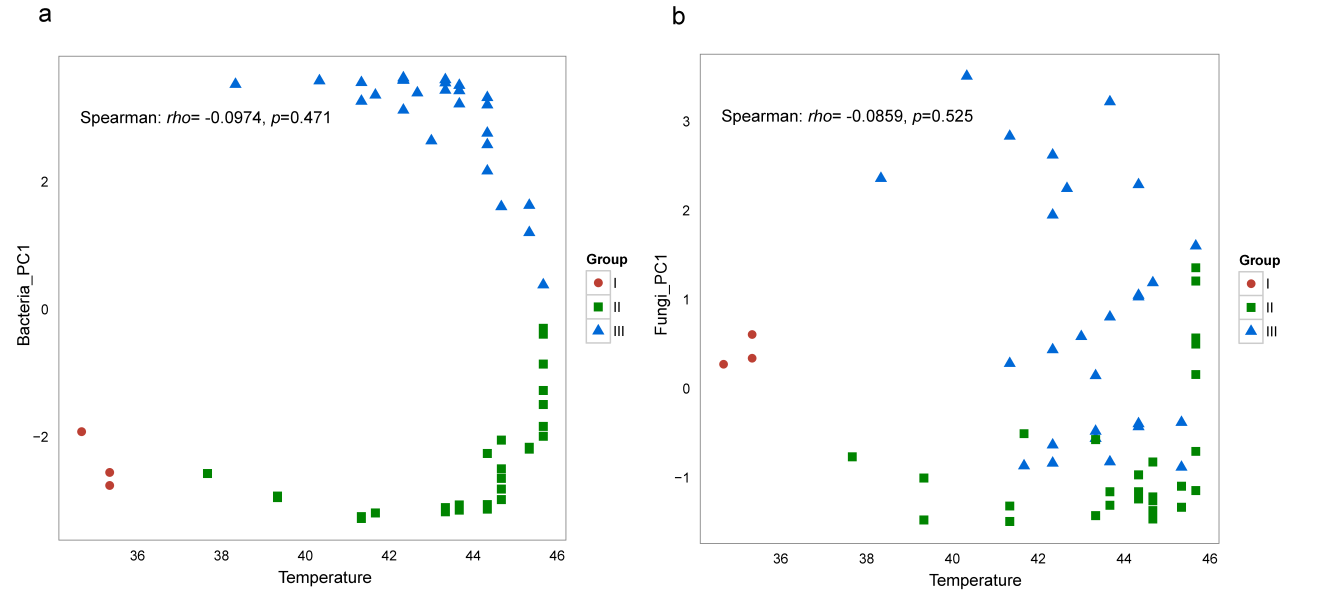


**Figure S4.** Correlation between the first principal components of **(a)** bacteria and **(b)** fungi and temperature.

**
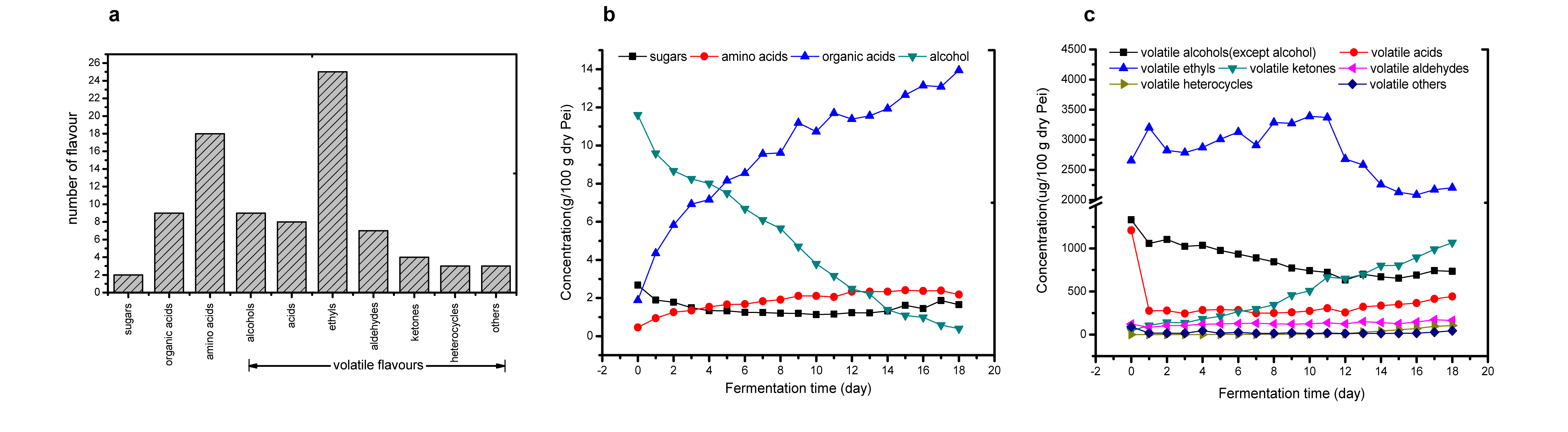
**

**Figure S5.** Detected flavours in vinegar *Pei* during AAF process of Zhenjiang aromatic vinegar.


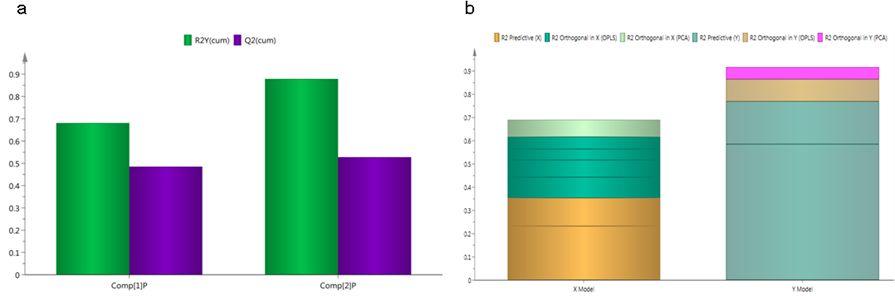


**Figure S6.** Summary of fit and overview plots for O2PLS model. **(a)** The summary fit plot displays the cumulative *R^2^* and *Q^2^* for the *Y*-matrix modeled by *X*. The labeled *P* in *x-*axis represents for predictive components. **(b)** The overview plot provides a graphical summary of the predictive and orthogonal sources of variation in the O2PLS model. The column is both stacked and color coded in accordance with the predictive and orthogonal variation structure.


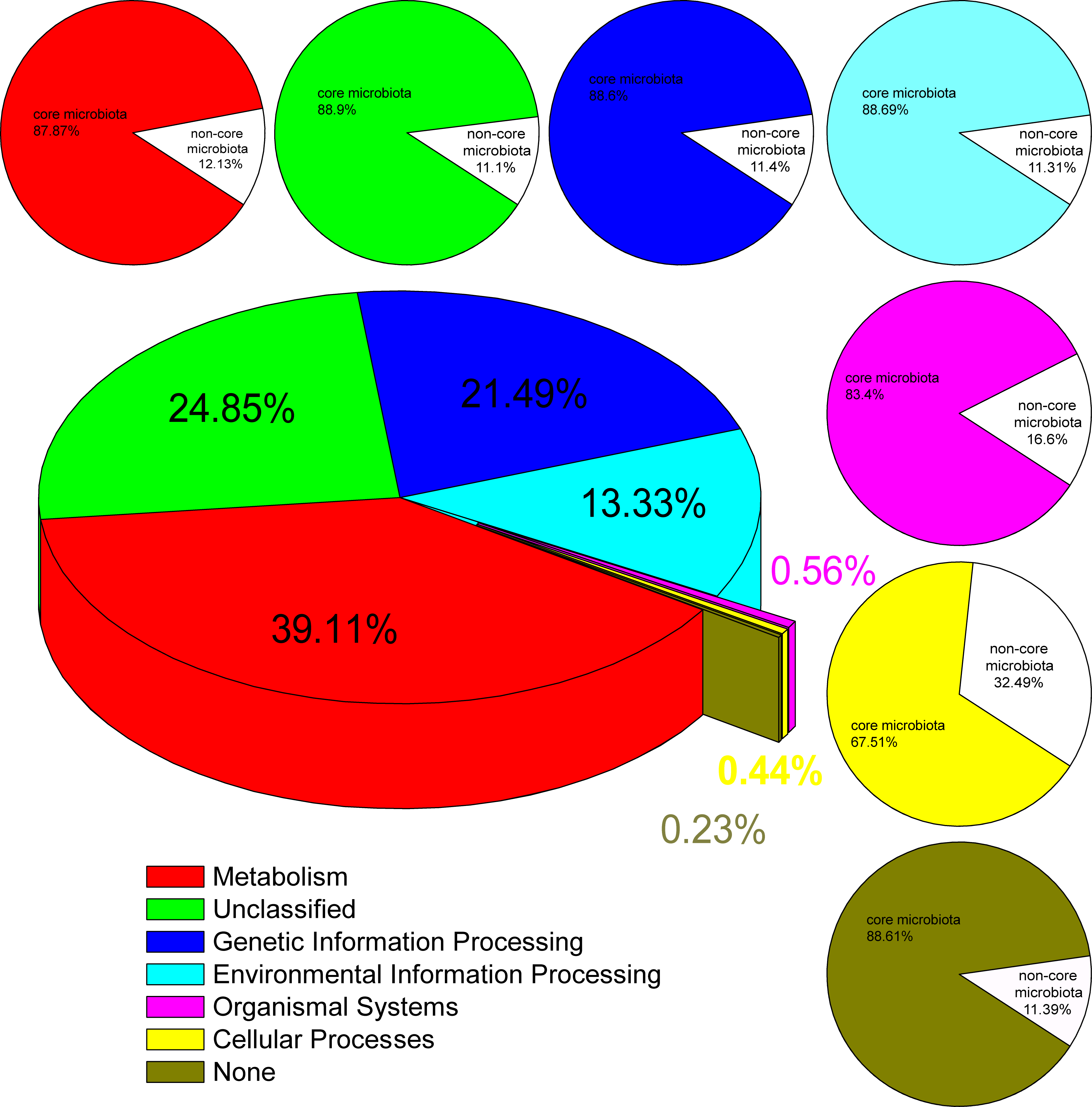


**Figure S7.** Predicted functions of the core microbiota and non-core microbiota in vinegar *Pei* by PICRUSt analysis.


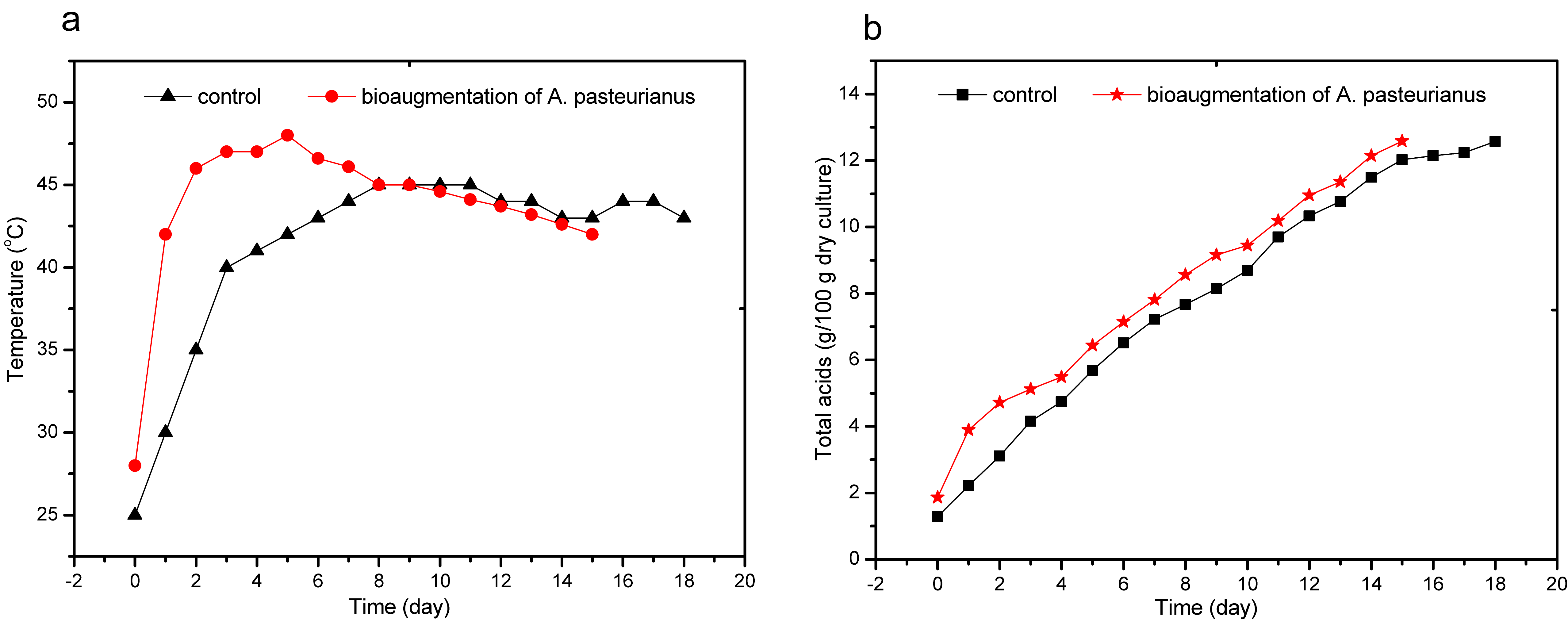


**Figure S8.** Changes of temperature **(a)** and total acids **(b)** in AAF process bioaugmented with *Acetobacter pasteurianus*.

**Supplementary table legends**

**Table S1.** The significantly different OTUs in bacteria between different stages of AAF.

**Table S2.** The significantly different OTUs in fungi between different stages of AAF.

**Table S3.** The distribution of flavours in different stages of AAF.

**Table S4.** The important microbiota correlated with all organic aicds (OAs), amino acids (AAs) and volatile flavors (VFs) (|*ρ*|>0.7).

**Table S5.** The important microbiota correlated with each of organic acid during AAF process (|*ρ*|>0.7).

**Table S6.** The important microbiota correlated with each of amino acid during AAF process (|*ρ*|>0.7).

**Table S7.** The important microbiota correlated with each of volatile flavour during AAF process (|*ρ*|>0.7).

**Table S8.** The number of flavours correlated with each microbe during AAF process (|*ρ*|>0.7).

**Table S9.** The number of flavours highly correlated (|*ρ*|>0.8) with each microbe during AAF process.

**Table S10.** Detrailed information of the functional core microbiota in vinegar *Pei* during AAF process.

**Table S11.** Comparsion of flavours between control batch and bioaugmentation of *A. pasteurianus*.

**Table S12.** Primers used for Miseq and biomass analysis in this study.

**Supplementary tables**

**Table S1.** The significantly different OTUs in bacteria between different stages of AAF.

| Group | No. of OTUs | OTU |
| --- | --- | --- |
| I  (0 d) | 38 | OTU3, OTU19, OTU21, OTU26, OTU27, OTU29, OTU37, OTU39, OTU60, OTU76, OTU78, OTU80, OTU95, OTU108, OTU124, OTU126, OTU131, OTU132, OTU136, OTU138, OTU144, OTU148, OTU157, OTU179, OTU185, OTU188, OTU189, OTU195, OTU197, OTU199, OTU208, OTU234, OTU241, OTU243, OTU245, OTU246, OTU264, OTU266, |
| II  (1-9 d) | 29 | OTU1, OTU21, OTU23, OTU26, OTU27, OTU37, OTU60, OTU78, OTU80, OTU91, OTU124, OTU125, OTU126, OTU127, OTU131, OTU132, OTU136, OTU138, OTU144, OTU149, OTU157, OTU188, OTU189, OTU197, OTU199, OTU241, OTU243, OTU245, OTU249, |
| III  (10-18d) | 52 | OTU11, OTU21, OTU26, OTU27, OTU33, OTU37, OTU60, OTU63, OTU65, OTU66, OTU72, OTU78, OTU80, OTU84, OTU90, OTU97, OTU107, OTU116, OTU123, OTU124, OTU126, OTU128, OTU131, OTU132, OTU136, OTU138, OTU140, OTU141, OTU144, OTU157, OTU162, OTU178, OTU180, OTU188, OTU189, OTU196, OTU197, OTU199, OTU203, OTU207, OTU211, OTU215, OTU217, OTU239, OTU241, OTU243, OTU244, OTU245, OTU254, OTU257, OTU262, OTU265, |

**Table S2.** The significantly different OTUs in fungi between different stages of AAF.

| Group | No. of OTUs | OTU |
| --- | --- | --- |
| I  (0 d) | 40 | OTU108, OTU114, OTU132, OTU142, OTU176, OTU187, OTU194, OTU219, OTU229, OTU232, OTU241, OTU252, OTU277, OTU296, OTU32, OTU341, OTU352, OTU355, OTU371, OTU377, OTU395, OTU409, OTU410, OTU446, OTU475, OTU490, OTU495, OTU498, OTU513, OTU54, OTU582, OTU59, OTU592, OTU594, OTU607, OTU617, OTU620, OTU622, OTU78, OTU83 |
| II  (1-9 d) | 29 | OTU101, OTU114, OTU132, OTU142, OTU176, OTU192, OTU194, OTU219, OTU22, OTU229, OTU23, OTU241, OTU282, OTU296, OTU32, OTU355, OTU395, OTU405, OTU458, OTU497, OTU513, OTU574, OTU578, OTU582, OTU59, OTU594, OTU603, OTU622, OTU83 |
| III  (10-18 d) | 25 | OTU114, OTU132, OTU142, OTU176, OTU194, OTU211, OTU219, OTU229, OTU241, OTU296, OTU32, OTU33, OTU355, OTU395, OTU425, OTU470, OTU513, OTU515, OTU582, OTU59, OTU594, OTU622, OTU646, OTU67, OTU83 |

**Table S3.** The distribution of flavours in different stages of AAF.

| Group | No. of flavours | Label of flavours | Name of flavours |
| --- | --- | --- | --- |
| 1  (day 0) | 13 | Fruc, Gluc, No.2, No.9, No.12, No.14, No.15, No.16, No.19, No.31, No.47, No.57, No.59, | fructose, glucose, 2-methyl-1-propanol, phenylethyl alcohol, pentanoic acid, hexanoic acid, heptanoic acid, octanoic acid, isobutyl acetate, hexyl hexanoate, hexanal, methoxy-phenyl-oxime, 4-methyl-phenol |
| 2  (days 1-7) | 15 | No.1, No.3, No.4, No.5, No.17, No.21, No.22, No.25, No.26, No.32, No.33, No.34, No.37, No.40, No.51, | alcohol, 3-methyl-1-butanol, 1-pentanol, 1-hexanol, azelaic acid, ethyl valerate, ethyl caproate, ethyl caprylate, isobutyl lactate, ethyl caprate, benzoic acid methyl ester, diethyl succinate, octyl caproate, 2-phenethyl hexanoate, 2-hydroxy-4-methyl-benaldehyde, |
| 3  (days 8-18) | 60 | AA, CA, LA, OA, PA, PgA, SA, KgA, TA, Ala, Arg, Asp, Cys, Gaba, Glu, Gly, His, Ile, Leu, Lys, Met, Phe, Pro, Ser, Thr, Tyr, Val, No.6, No.7, No.8, No.10, No.11, No.13, No.18, No.20, No.23, No.24, No.27, No.28, No.29, No.30, No.35, No.36, No.38, No.39, No.41, No.42, No.43, No.44, No.45, No.46, No.48, No.49, No.50, No.52, No.53, No.54, No.55, No.56, No.58, | acetic acid, citric acid, lactic acid, oxalic acid, pyruvic acid, pyroglutamic acid, succinic acid, tartaric acid, alanine, arginine, aspartic acid, cysteine, γ-aminobutyric acid, glutamic acid, glycine, histidine, isoleucine, ketoglutaric acid, leucine, lysine, methionine, phenylalanine, proline, serine, threonine, tyrosine, valine, 2,3-butanediol, 2-furanmethanol, beta-ethylphenethyl alcohol, 2-ethyl-2-hydroxybutyric acid, 2-methyl-propanoic acid, 3-methyl-butanoic acid, ethyl acetate, isopentyl acetate, ethyl lactate, 3-hydroxy-2-butanone-acetate, furfuryl acetate, 2-hydroxy-4-methyl-ethyl valerate, isoamyl lactate, 3-methylthio-propionate ether, ethyl benzoate, ethyl phenylacetate, phenethyl acetate, ethyl laurate, ethyl palmitate, ethyl oleate, 2,3-butanedione, 3-hydroxy-2-butanone, 1-phenyl-1-propanone, 5-heptyldihydro-2(3H)-furanone, nonanal, furfural, benzaldehyde, alpha-ethylidene-phenylacetaldehyde, 4-pentyl-benzaldehyde, trimethyl-oxazole, 2,3-dimethyl-pyrazine, 2,3,5,6-tetramethyl-pyrazine, 2-methoxy-4-methyl-phenol, |

**Table S4.** The important microbiota correlated with all organic aicds (OAs), amino acids (AAs) and volatile flavours (VFs) (|*ρ*|>0.7).

| Flavor | No. of genus | Microbiota in genus^*a^ |
| --- | --- | --- |
| AAs | 59  (48B^*b^+11F^*c^) | *Acetobacter, Aequorivita, Alkaliphilus, Arthrobacter, Aurantimonas, Bacillales_unclassified, Bacillus, Curtobacterium, Bacteria_unclassified, Brochothrix, Burkholderiales_incertae_sedis_unclassified, Carnobacterium, Corynebacterium, Comamonadaceae_unclassified, Dietzia, Enhydrobacter, Enterococcus, Exiguobacterium, Flavobacterium, Massilia, Gluconacetobacter, Halomonas, Kineococcus, Lactobacillus, Lactococcus, Leuconostoc, Luteibacter, Pedobacter, Methylobacterium, Mucilaginibacter, Nitriliruptor, Novosphingobium, Paracoccus, Planococcaceae_unclassified, Propionibacterium, Proteobacteria_unclassified, Rhizobium, Roseomonas, Ruminococcaceae_unclassified , Sphingobacterium, Sphingobium, Sphingomonas, Staphylococcus, Stenotrophomonas, Streptococcus, Thermomonas, Weissella, Xanthomonas, Alternaria, Aspergillus, Epicoccum, Eurotiales_unclassified, Fungi_unclassified, Malassezia, Verticillium, Mycosphaerellaceae_unclassified, Phaeoacremonium, Pseudozyma, Thermomyces* |
| OAs | 47  (36B+11F) | *Acetobacter, Aequorivita, Arthrobacter, Bacillales_unclassified, Bacillus, Bacteria_unclassified, Rhizobium, Brochothrix, Enhydrobacter, Enterococcus, Burkholderiales_incertae_sedis_unclassified, Carnobacterium, Comamonadaceae_unclassified, Corynebacterium, Exiguobacterium, Flavobacterium, Gluconacetobacter, Halomonas, Lactobacillus, Lactococcus, Luteibacter, Massilia, Methylobacterium, Nitriliruptor, Sphingobium, Novosphingobium, Planococcaceae_unclassified, Proteobacteria_unclassified, Pseudomonas, Roseomonas, Streptococcus, Weissella, Ruminococcaceae_unclassified, Sphingobacterium, Staphylococcus, Stenotrophomonas, Alternaria, Aspergillus, Cystobasidiomycetes_unclassified, Fungi_unclassified, Fusarium, Malassezia, Phaeoseptoria, Pleosporales_unclassified, Sarocladium, Sterigmatomyces, Verticillium,* |
| VFs | 92  (61B+31F) | *Acetobacter, Acidovorax, Aequorivita, Alcaligenaceae_unclassified, Alkaliphilus, Aquabacterium, Arcicella, Arthrobacter, Aurantimonas, Bacillales_unclassified, Bacillus, Bacteria_unclassified, Brochothrix, Kineococcus, Cupriavidus, Burkholderiales_incertae_sedis_unclassified,, Carnobacterium, Gelidibacter, Comamonadaceae_unclassified, Corynebacterium, Curtobacterium,, Dietzia, Enhydrobacter, Enterococcus, Exiguobacterium, Flavobacterium, Sphingobium, Gluconacetobacter, Halomonas, Lactobacillus, Lactococcus, Leuconostoc, Luteibacter, Massilia, Methylobacterium, Mucilaginibacter, Nitriliruptor, Novosphingobium, Ochrobactrum, Paracoccus, Pedobacter, Xanthomonas, Weissella, Planococcaceae_unclassified, Propionibacterium, Sphingomonas, Proteobacteria_unclassified Pseudomonas, Rhizobium, Roseomonas, Ruminococcaceae_unclassified, Saccharopolyspora, Sphingobacterium, Sphingopyxis, Staphylococcus, Stenotrophomonas, Streptococcus, Thermomonas, unclassified_Actinomycetales_norank, Veillonella, Wautersiella, Actinomucor, Alternaria, Ascomycota_unclassified, Aspergillus, Cryptococcus, Cystobasidiomycetes_unclassified, Epicoccum, Erythrobasidium, Eurotiales_unclassified, Filobasidiaceae_unclassified, Fungi_unclassified, Fusarium, Leptosphaeria, Malassezia, Meyerozyma, Microdochium, Monographella, Phaeoseptoria, Phialosimplex, Verticillium, Pleosporaceae_unclassified, Pleosporales_unclassified, Pseudozyma, Pyrenochaetopsis, Rhizomucor, Sarocladium, Sterigmatomyces, Thermomyces, Tremellomycetes_unclassified, Trichosphaeriales_unclassified, Unidentified,* |

**a-- Black font represents bacteria and green font represents fungi. *b--The character B is the abbreviation of bacteria. *c-- The character F is the abbreviation of fungi.*

**Table S5.** The important microbiota correlated with each of organic acid during AAF process (|*ρ*|>0.7).

| Organic acids | Microbes in genus^*a^ (positive correlation: *ρ*>0.7 ) | Microbes in genus^*a^ (negative correlation: |*ρ*|>0.7 ) |
| --- | --- | --- |
| Oxalic acid  (17) (14B^*b^+3F^*c^) | *Acetobacter, Enhydrobacter, Lactococcus, Bacillales_unclassified, Bacillus, Enterococcus, Gluconacetobacter, Arthrobacter, Aspergillus, Verticillium,* | *Luteibacter, Stenotrophomonas, Roseomonas, Sphingobacterium, Lactobacillus, Staphylococcus, Fungi_unclassified,* |
| Tartaric acid  (19) (17B+2F) | *Gluconacetobacter, Acetobacter, Bacillus, Lactococcus, Enhydrobacter, Nitriliruptor, Ruminococcaceae_unclassified, Sphingobium, Bacillales_unclassified, Corynebacterium, Planococcaceae_unclassified, Halomonas, Enterococcus, Streptococcus, Arthrobacter, Brochothrix, Verticillium* | *Lactobacillus, Alternaria,* |
| Pyruvic acid  (23) (21B+2F) | *Gluconacetobacter, Acetobacter, Lactococcus, Enhydrobacter, Halomonas, Brochothrix, Bacillales_unclassified, Bacillus, Planococcaceae_unclassified, Exiguobacterium, Ruminococcaceae_unclassified, Enterococcus, Sphingobium, Arthrobacter, Nitriliruptor, Carnobacterium, Streptococcus, Aequorivita, Burkholderiales_incertae_sedis_unclassified, Corynebacterium, Verticillium, Sterigmatomyces* | *Lactobacillus* |
| Ketoglutaric acid  (14) (11B+3F) | *Gluconacetobacter, Ruminococcaceae_unclassified, Sphingobium, Bacillus, Streptococcus, Lactococcus, Planococcaceae_unclassified, Enhydrobacter, Bacillales_unclassified, Enterococcus,* | *Lactobacillus, Cystobasidiomycetes_unclassified, Sarocladium,*  *Pleosporales_unclassified,* |

| Lactic acid  (16) (13B+3F) | *Phaeoseptoria,*  *Fusarium,* | *Roseomonas, Rhizobium, Novosphingobium, Luteibacter, Weissella, Methylobacterium, Massilia, Staphylococcus, Pseudomonas, Comamonadaceae_unclassified, Bacteria_unclassified, Sphingobacterium, Proteobacteria_unclassified, Fungi_unclassified,* |
| --- | --- | --- |
| Acetic acid  (25) (21B+4F) | *Acetobacter, Enhydrobacter, Lactococcus, Gluconacetobacter, Arthrobacter, Sphingobium, Bacillales_unclassified, Bacillus, Enterococcus, Exiguobacterium, Streptococcus, Aequorivita, Planococcaceae_unclassified, Carnobacterium, Nitriliruptor, Flavobacterium, Halomonas, Brochothrix, Verticillium, Aspergillus, Ruminococcaceae_unclassified, Burkholderiales_incertae_sedis_unclassified, Malassezia,* | *Lactobacillus,*  *Alternaria* |
| Citric acid (7) (7B+0F) | *Acetobacter, Gluconacetobacter, Enhydrobacter,* | *Roseomonas, Sphingobacterium, Staphylococcus, Lactobacillus,* |
| Pyroglutamic acid  (24) (21B+3F) | *Acetobacter, Enhydrobacter, Lactococcus, Gluconacetobacter, Aequorivita, Arthrobacter,*  *Bacillales_unclassified, Bacillus, Enterococcus, Streptococcus, Sphingobium, Halomonas, arnobacterium, Planococcaceae_unclassified, Brochothrix, Nitriliruptor, Exiguobacterium, Ruminococcaceae_unclassified, Burkholderiales_incertae_sedis_unclassified, Corynebacterium, Verticillium, Aspergillus* | *Lactobacillus, Alternaria* |
| Succinic acid  (24) (22B+2F) | *Gluconacetobacter, Acetobacter, Lactococcus, Enhydrobacter, Arthrobacter, Streptococcus, Ruminococcaceae_unclassified, Sphingobium, Bacillales_unclassified, Bacillus, Enterococcus, Halomonas, Planococcaceae_unclassified, Brochothrix, Corynebacterium, Carnobacterium, Aequorivita, Nitriliruptor, Exiguobacterium, Flavobacterium, Verticillium, Burkholderiales_incertae_sedis_unclassified, Sterigmatomyces,* | *Lactobacillus* |

**a-- Black font represents bacteria and green font represents fungi. *b--The character B is the abbreviation of bacteria. *c-- The character F is the abbreviation of fungi.*

**Table S6.** The important microbiota correlated with each of amino acid during AAF process (|*ρ*|>0.7).

| Amino acids | Microbes in genus^*a^ (positive correlation: *ρ*>0.7 ) | Microbes in genus^*a^ (negative correlation: |*ρ*|>0.7 ) |
| --- | --- | --- |
| Aspartic acid  (16) (12B^*a^+4F^*b^) | *Acetobacter, Enhydrobacter, Lactococcus, Bacillus, Arthrobacter,*  *Bacillales_unclassified, Enterococcus, Aspergillus, Verticillium, Eurotiales_unclassified,* | *Stenotrophomonas, Roseomonas, Lactobacillus, Sphingobacterium, Staphylococcus, Fungi_unclassified* |
| Glutamic acid  (14) (11B+3F) | *Acetobacter, Enhydrobacter,*  *Aspergillus, Eurotiales_unclassified,* | *Bacteria_unclassified, Rhizobium, Weissella, Lactobacillus, Sphingobacterium, Stenotrophomonas, Luteibacter, Roseomonas, Staphylococcus, Fungi_unclassified* |
| Serine  (28) (25B+3F) | *Brochothrix, Bacillus, Lactococcus, Dietzia, Gluconacetobacter, Acetobacter, Enterococcus, Alkaliphilus, Bacillales_unclassified, Streptococcus, Flavobacterium, Planococcaceae_unclassified, Thermomonas, Paracoccus, Exiguobacterium, Halomonas, Enhydrobacter, Arthrobacter, Sphingobium, Ruminococcaceae_unclassified, Leuconostoc,Carnobacterium, Corynebacterium, Verticillium, Malassezia, Phaeoacremonium, Burkholderiales_incertae_sedis_unclassified,* | *Lactobacillus* |
| Histidine  (26) (23B+3F) | *Enhydrobacter, Bacillus, Lactococcus, Gluconacetobacter, Streptococcus, Bacillales_unclassified, Enterococcus, Propionibacterium, Arthrobacter, Flavobacterium, Planococcaceae_unclassified, Exiguobacterium, Halomonas, Brochothrix, Sphingobium, Carnobacterium, Acetobacter, Dietzia, Burkholderiales_incertae_sedis_unclassified, Alkaliphilus, Leuconostoc, Ruminococcaceae_unclassified, Verticillium, Malassezia, Pseudozyma,* | *Lactobacillus* |
| Glycine  (20) (17B+3F) | *Acetobacter, Enhydrobacter,*  *Aspergillus, Eurotiales_unclassified* | *Proteobacteria_unclassified, Stenotrophomonas, Methylobacterium, Weissella, Comamonadaceae_unclassified, Aurantimonas, Roseomonas, Luteibacter, Sphingomonas, Rhizobium, Mucilaginibacter, Lactobacillus, Sphingobacterium, Bacteria_unclassified, Staphylococcus, Fungi_unclassified,* |

| Threonine  (21) (18B+3F) | *Acetobacter, Enhydrobacter,*  *Aspergillus, Eurotiales_unclassified* | *Pedobacter, Proteobacteria_unclassified, Weissella, Methylobacterium, Luteibacter, Aurantimonas, Sphingomonas, Comamonadaceae_unclassified, Rhizobium, Mucilaginibacter, Lactobacillus, Bacteria_unclassified, Staphylococcus, Stenotrophomonas, Roseomonas, Sphingobacterium, Fungi_unclassified* |
| --- | --- | --- |
| Arginine  (26) (22B+4F) | *Acetobacter, Enhydrobacter, Lactococcus, Enterococcus, Bacillus, Gluconacetobacter, Bacillales_unclassified, Arthrobacter, Streptococcus, Carnobacterium, Sphingobium, Exiguobacterium, Flavobacterium, Planococcaceae_unclassified, Brochothrix, Nitriliruptor, Alkaliphilus,*  *Ruminococcaceae_unclassified, Halomonas, Aequorivita, Malassezia, Burkholderiales_incertae_sedis_unclassified, Verticillium, Aspergillus,* | *Lactobacillus,*  *Alternaria* |
| Alanine  (16) (13B+3F) | *Acetobacter, Enhydrobacter, Lactococcus, Arthrobacter, Enterococcus, Bacillales_unclassified, Bacillus, Aspergillus, Eurotiales_unclassified,* | *Luteibacter, Stenotrophomonas, Roseomonas, Sphingobacterium, Lactobacillus, Staphylococcus, Fungi_unclassified,* |
| γ-aminobutyric acid  (7) (3B+4F) | *Massilia, Streptococcus, Mycosphaerellaceae_unclassified* | *Lactobacillus, Thermomyces, Alternaria, Epicoccum,* |
| Tyrosine  (10) (7B+3F) | *Acetobacter,*  *Aspergillus, Eurotiales_unclassified* | *Stenotrophomonas, Weissella, Luteibacter, Roseomonas, Sphingobacterium, Staphylococcus, Fungi_unclassified,* |
| Cysteine  (22) (19B+3F) | *Aspergillus,*  *Eurotiales_unclassified,* | *Curtobacterium, Bacteria_unclassified, Rhizobium, Roseomonas, Kineococcus, Stenotrophomonas, Xanthomonas, Novosphingobium, Luteibacter, Staphylococcus, Proteobacteria_unclassified, Comamonadaceae_unclassified, Pedobacter, Methylobacterium, Mucilaginibacter, Weissella Sphingomonas, Sphingobacterium, Aurantimonas, Fungi_unclassified,* |
| Valine  (14) (11B+3F) | *Acetobacter, Enhydrobacter, Lactococcus, Bacillales_unclassified, Aspergillus, Eurotiales_unclassified,* | *Weissella, Luteibacter, Stenotrophomonas, Lactobacillus, Roseomonas, Sphingobacterium, Staphylococcus, Fungi_unclassified* |

| Methionine  (13) (10B+3F) | *Acetobacter,*  *Aspergillus, Eurotiales_unclassified,* | *Comamonadaceae_unclassified, Staphylococcus, Proteobacteria_unclassified, Sphingobacterium,Bacteria_unclassified, Stenotrophomonas, Luteibacter, Weissella, Roseomonas, Fungi_unclassified,* |
| --- | --- | --- |
| Phenylalanine  (20) (17B+3F) | *Eurotiales_unclassified,*  *Aspergillus,* | *Xanthomonas, Novosphingobium, Sphingomonas, Methylobacterium, Comamonadaceae_unclassified, Mucilaginibacter, Aurantimonas, Rhizobium, Pedobacter, Proteobacteria_unclassified, Weissella, Bacteria_unclassified, Luteibacter, Roseomonas, Stenotrophomonas, Sphingobacterium, Staphylococcus, Fungi_unclassified,* |
| Isoleucine  (13) (10B+3F) | *Acetobacter, Enhydrobacter,*  *Aspergillus, Eurotiales_unclassified,* | *Bacteria_unclassified, Weissella, Lactobacillus, Luteibacter, Staphylococcus, Stenotrophomonas, Roseomonas, Sphingobacterium, Fungi_unclassified,* |
| Leucine  (22) (19B+3F) | *Acetobacter,*  *Enhydrobacter,*  *Aspergillus,*  *Eurotiales_unclassified,* | *Xanthomonas, Proteobacteria_unclassified, Comamonadaceae_unclassified, Methylobacterium, Mucilaginibacter, Aurantimonas, Lactobacillus, Sphingomonas, Pedobacter, Rhizobium, Weissella,Bacteria_unclassified, Stenotrophomonas, Luteibacter, Roseomonas, Sphingobacterium, Staphylococcus, Fungi_unclassified,* |
| Lysine  (24) (19B+5F) | *Enhydrobacter, Acetobacter, Lactococcus, Enterococcus, Streptococcus, Bacillales_unclassified, Bacillus, Arthrobacter, Aequorivita, Sphingobium, Gluconacetobacter, Carnobacterium, Halomonas, Exiguobacterium, Brochothrix, Nitriliruptor, Malassezia, Planococcaceae_unclassified, Ruminococcaceae_unclassified, Verticillium, Aspergillus,* | *Lactobacillus,*  *Thermomyces,*  *Alternaria,* |
| Proline  (14) (12B+2F) | *Acetobacter,*  *Enhydrobacter,*  *Aspergillus,* | *Comamonadaceae_unclassified, Mucilaginibacter, Bacteria_unclassified, Weissella, Lactobacillus, Luteibacter, Stenotrophomonas, Roseomonas, Sphingobacterium, Staphylococcus, Fungi_unclassified* |

**a-- Black font represents bacteria and green font represents fungi. *b--The character B is the abbreviation of bacteria. *c-- The character F is the abbreviation of fungi.*

**Table S7.** The important microbiota correlated with each of volatile flavour during AAF process (|*ρ*|>0.7).

| Valitile flavour | Microbes in genus^*a^ (positive correlation: *ρ*>0.7 ) | Microbes in genus^*a^ (negative correlation: |*ρ*|>0.7 ) |
| --- | --- | --- |
| No.1  (22) (19B^*b^+3F^*c^) | *Lactobacillus, Staphylococcus, Alternaria,* | *Brochothrix, Nitriliruptor, Bacillales_unclassified, Exiguobacterium, Halomonas, Burkholderiales_incertae_sedis_unclassified, Sphingobium, Bacillus, Acetobacter, Planococcaceae_unclassified, Streptococcus, Carnobacterium, Gluconacetobacter, Arthrobacter, Enterococcus, Lactococcus, Enhydrobacter, Verticillium, Aspergillus,* |
| No.2  (23) (21B+2F) | *Staphylococcus, Weissella, Luteibacter, Stenotrophomonas, Bacteria_unclassified, Rhizobium, Methylobacterium, Proteobacteria_unclassified, Sphingomonas, Roseomonas, Comamonadaceae_unclassified, Sphingobacterium, Aurantimonas, Mucilaginibacter, Novosphingobium, Pedobacter, Curtobacterium, Xanthomonas, Pseudomonas,*  *Alcaligenaceae_unclassified, Fungi_unclassified,* | *Acetobacter, Aspergillus,* |
| No.3  (19) (16B+3F) | *Lactobacillus, Staphylococcus, Stenotrophomonas, Roseomonas, Sphingobacterium,*  *Luteibacter, Fungi_unclassified,* | *Nitriliruptor, Carnobacterium, Gluconacetobacter, Arthrobacter, Bacillus, Enterococcus, Acetobacter,Bacillales_unclassified, Lactococcus, Enhydrobacter, Verticillium, Aspergillus,* |
| No.4  (17) (15B+2F) | *Lactobacillus, Staphylococcus,* | *Sphingobium, Streptococcus, Gluconacetobacter, Planococcaceae_unclassified, Arthrobacter, Bacillus, Nitriliruptor, Carnobacterium, Enterococcus, Acetobacter, Bacillales_unclassified, Lactococcus, Enhydrobacter, Verticillium, Aspergillus,* |
| No.5  (29) (24B+5F) | *Lactobacillus,*  *Alternaria,*  *Epicoccum,*  *Pleosporales_unclassified,* | *Thermomonas, Aequorivita, Alkaliphilus, Leuconostoc, Brochothrix, Nitriliruptor, Bacillales_unclassified, Burkholderiales_incertae_sedis_unclassified, Halomonas, Flavobacterium, Exiguobacterium, Ruminococcaceae_unclassified, Arthrobacter,*  *Planococcaceae_unclassified, Carnobacterium, Sphingobium, Streptococcus, Gluconacetobacter, Enterococcus, Enhydrobacter, Bacillus, Acetobacter, Lactococcus, Malassezia, Verticillium,* |
| No.6  (23) (22B+1F) | *Acetobacter, Gluconacetobacter, Enhydrobacter, Paracoccus, Lactococcus, Nitriliruptor, Bacillales_unclassified, Bacillus, Sphingobium, Enterococcus, Halomonas, Arthrobacter, Exiguobacterium, Ruminococcaceae_unclassified, Streptococcus, Planococcaceae_unclassified, Brochothrix, Aequorivita, Corynebacterium, Flavobacterium, Carnobacterium, Verticillium,* | *Lactobacillus* |

| No.7  (24) (23B+1F) | *Acetobacter, Enhydrobacter, Gluconacetobacter, Arcicella, Lactococcus, Bacillus, Bacillales_unclassified, Halomonas, Enterococcus, Arthrobacter, Streptococcus, Aequorivita, Planococcaceae_unclassified, Sphingobium, Nitriliruptor, Brochothrix, Corynebacterium, Ruminococcaceae_unclassified, Carnobacterium, Exiguobacterium, Verticillium, Aspergillus,* | *Lactobacillus, Alternaria,* |
| --- | --- | --- |
| No.8  (23) (21B+2F) | *Gluconacetobacter, Acetobacter, Lactococcus, Enhydrobacter, Bacillus, Bacillales_unclassified, Enterococcus, Streptococcus, Sphingobium, Arthrobacter, Halomonas, Nitriliruptor, Carnobacterium, Planococcaceae_unclassified, Ruminococcaceae_unclassified, Leuconostoc, Exiguobacterium, Paracoccus, Brochothrix, Flavobacterium, Meyerozyma, Verticillium,* | *Lactobacillus* |
| No.9  (3) (1B+2F) | *----* | *Acetobacter, Aspergillus, Eurotiales_unclassified* |
| No.10  (20) (18B+2F) | *Acetobacter, Enhydrobacter, Lactococcus, Bacillales_unclassified, Enterococcus, Gluconacetobacter, Arthrobacter, Halomonas, Streptococcus, Exiguobacterium,*  *Planococcaceae_unclassified, Brochothrix, Carnobacterium, Flavobacterium, Ruminococcaceae_unclassified, Sphingobium, Bacillus, Verticillium, Aspergillus,* | *Lactobacillus* |
| No.11  (23) (21B+2F) | *Acetobacter, Gluconacetobacter, Enhydrobacter, Gelidibacter, Lactococcus, Bacillus, Bacillales_unclassified, Enterococcus, Planococcaceae_unclassified, Arthrobacter, Exiguobacterium, Carnobacterium, Streptococcus , Sphingobium, Nitriliruptor,*  *Ruminococcaceae_unclassified, Flavobacterium, Brochothrix, Halomonas, Burkholderiales_incertae_sedis_unclassified, Verticillium, Aspergillus* | *Lactobacillus* |
| No.12  (23) (21B+2F) | *Bacteria_unclassified, Weissella, Proteobacteria_unclassified, Rhizobium, Mucilaginibacter, Sphingobacterium, Acidovorax, Comamonadaceae_unclassified, Luteibacter, Methylobacterium, Novosphingobium, Sphingomonas, Aurantimonas, Stenotrophomonas, Staphylococcus, Roseomonas, Pseudomonas, Pedobacter, Xanthomonas, Alcaligenaceae_unclassified, Curtobacterium, Fungi_unclassified* | *Aspergillus,* |

| No.13  (26) (24B+2F) | *Gluconacetobacter, Lactococcus, Acetobacter, Enhydrobacter, Enterococcus, Bacillales_unclassified, Bacillus, Arthrobacter, Planococcaceae_unclassified, Streptococcus, Sphingobium, Ruminococcaceae_unclassified, Carnobacterium, Brochothrix, Halomonas, Exiguobacterium, Leuconostoc, Aequorivita, Burkholderiales_incertae_sedis_unclassified, Flavobacterium, Corynebacterium, Alkaliphilus, Nitriliruptor, Verticillium, Malassezia,* | *Lactobacillus* |
| --- | --- | --- |
| No.14  (22) (21B+2F) | *Proteobacteria_unclassified, Bacteria_unclassified, Weissella, Rhizobium, Luteibacter, Comamonadaceae_unclassified, Novosphingobium, Methylobacterium, Mucilaginibacter, Pseudomonas, Aurantimonas, Sphingomonas, Stenotrophomonas, Staphylococcus, Pedobacter, Alcaligenaceae_unclassified, Roseomonas, Xanthomonas, Curtobacterium, Acidovorax, Sphingobacterium, Fungi_unclassified,* |  |
| No.15  (22) (21B+2F) | *Proteobacteria_unclassified, Bacteria_unclassified, Weissella, Rhizobium, Novosphingobium, Comamonadaceae_unclassified, Acidovorax, Methylobacterium, Pseudomonas, Curtobacterium, Mucilaginibacter, Luteibacter, Aurantimonas, Sphingomonas, Staphylococcus, Roseomonas, Sphingobacterium, Stenotrophomonas, Pedobacter, Alcaligenaceae_unclassified, Xanthomonas, Fungi_unclassified,* |  |
| No.16  (22) (21B+2F) | *Proteobacteria_unclassified, Bacteria_unclassified, Weissella, Rhizobium, Luteibacter, Comamonadaceae_unclassified, Curtobacterium, Methylobacterium, Novosphingobium , Sphingobacterium, Mucilaginibacter, Staphylococcus, Pedobacter, Sphingomonas, Aurantimonas, Pseudomonas, Roseomonas, Stenotrophomonas, Alcaligenaceae_unclassified, Fungi_unclassified,* |  |
| No.17  (14) (13B+1F) | *Lactobacillus,*  *Alternaria,* | *Arthrobacter, Carnobacterium, Bacillus, Lactococcus, Streptococcus, Leuconostoc, Planococcaceae_unclassified, Exiguobacterium, Gluconacetobacter, Enhydrobacter, Bacillales_unclassified, Enterococcus,* |
| No.18  (2) (1B+1F) | *Sphingopyxis, Aspergillus,* |  |

| No.19  (15) (13B+2F) | *Weissella, Pseudomonas, Proteobacteria_unclassified, Methylobacterium, Novosphingobium, Rhizobium, Acidovorax, Bacteria_unclassified, Aurantimonas, Sphingomonas, Luteibacter, Staphylococcus, Sphingobacterium,* | *Pleosporales_unclassified,*  *Rhizomucor,* |
| --- | --- | --- |
| No.20  (9) (6B+3F) | *Acetobacter, Sphingopyxis, Eurotiales_unclassified, Aspergillus,* | *Roseomonas, Comamonadaceae_unclassified, Staphylococcus, unclassified_Actinomycetales_norank, Fungi_unclassified,* |
| No.21  (21) (20B+1F) | *Lactobacillus, Sphingobacterium, Kineococcus, Roseomonas, Xanthomonas, Curtobacterium, Wautersiella, Staphylococcus,* | *Carnobacterium, Bacillus, Brochothrix, Enhydrobacter, Arthrobacter, Enterococcus, Gluconacetobacter, Planococcaceae_unclassified, Bacillales_unclassified, Lactococcus, Sphingobium, Ruminococcaceae_unclassified, Verticillium,* |
| No.22  (31) (29B+2F) | *Stenotrophomonas, Staphylococcus, Roseomonas, Sphingobacterium, Luteibacter, Xanthomonas, Weissella, Sphingomonas, Curtobacterium, Rhizobium, Aurantimonas, Mucilaginibacter, Lactobacillus, Pedobacter, Methylobacterium, Bacteria_unclassified, Fungi_unclassified,* | *Brochothrix, Carnobacterium, Bacillus, Planococcaceae_unclassified, Arthrobacter, Ruminococcaceae_unclassified, Sphingobium, Enterococcus, Enhydrobacter, Bacillales_unclassified, Acetobacter, Lactococcus, Gluconacetobacter, Verticillium,* |
| No.23  (19) (12B+7F) | *Pleosporales_unclassified, Sarocladium, Fusarium, Leptosphaeria,*  *Cystobasidiomyce,tes_unclassified,, Trichosphaeriales_unclassified,* | *Rhizobium, Comamonadaceae_unclassified, Staphylococcus, Novosphingobium, Aquabacterium, Methylobacterium, Pseudomonas, Acidovorax, Massilia, Bacteria_unclassified, Weissella, Proteobacteria_unclassified, Fungi_unclassified,* |
| No.24  (27) (24B+3F) | *Gluconacetobacter, Lactococcus, Acetobacter, Enhydrobacter, Bacillus, Arthrobacter, Bacillales_unclassified, Enterococcus, Paracoccus, Sphingobium, Flavobacterium, Ruminococcaceae_unclassified, Streptococcus, Planococcaceae_unclassified, Halomonas, Carnobacterium, Brochothrix, Nitriliruptor, Exiguobacterium, Corynebacterium, Aequorivita, Alkaliphilus, Sterigmatomyces, Burkholderiales_incertae_sedis_unclassified, Verticillium, Malassezia,* | *Lactobacillus* |
| No.25  (10) (8B+2F) | *Lactobacillus, Xanthomonas,* | *Veillonella, Lactococcus, Corynebacterium, Gluconacetobacter, Sphingobium,*  *Ruminococcaceae_unclassified, Pseudozyma, Verticillium,* |
| No.26  (9) (6B+3F) | *Lactobacillus, Pleosporales_unclassified, Alternaria, Sarocladium,* | *Lactococcus, Acetobacter, Nitriliruptor, Gluconacetobacter, Sphingobium,* |
| No.27  (16) (13B+3F) | *Gluconacetobacter, Ruminococcaceae_unclassified, Sphingobium, Acetobacter, Halomonas, Corynebacterium, Lactococcus, Nitriliruptor, Bacillus, Paracoccus, Enhydrobacter, Bacillales_unclassified, Phialosimplex, Cryptococcus, Sterigmatomyces,* | *Lactobacillus* |

| No.28  (18) (15B+3F) | *Lactobacillus,*  *Fusarium,*  *Sarocladium,* | *Exiguobacterium, Brochothrix, Carnobacterium, Arthrobacter, Lactococcus, Burkholderiales_incertae_sedis_unclassified, Planococcaceae_unclassified, Sphingobium, Enterococcus, Gluconacetobacter, Ruminococcaceae_unclassified, Bacillales_unclassified, Bacillus, Streptococcus, Malassezia,* |
| --- | --- | --- |
| No.29  (6) (4B+2F) | *Sarocladium, Pleosporales_unclassified,* | *Aquabacterium, Acidovorax, Massilia, Pseudomonas,* |
| No.30^*d^  (1) (1B+0F) | *----* | *Massilia* |
| No.31  (24) (22B+2F) | *Staphylococcus, Weissella, Luteibacter, Stenotrophomonas, Bacteria_unclassified, Rhizobium, Methylobacterium, Proteobacteria_unclassified, Mucilaginibacter, Aurantimonas, Comamonadaceae_unclassified, Sphingomonas, Roseomonas, Novosphingobium, Sphingobacterium, Pedobacter, Xanthomonas, Curtobacterium, Kineococcus,Pseudomonas, Alcaligenaceae_unclassified, Fungi_unclassified,* | *Acetobacter,*  *Aspergillus,* |
| No.32  (24) (23B+1F) | *Roseomonas, Staphylococcus, Stenotrophomonas, Sphingobacterium, Luteibacter, Xanthomonas, Mucilaginibacter, Sphingomonas, Aurantimonas, Rhizobium, Weissella, Methylobacterium, Curtobacterium, Bacteria_unclassified, Pedobacter, Novosphingobium, Wautersiella, Proteobacteria_unclassified, Kineococcus, Comamonadaceae_unclassified, Saccharopolyspora, Fungi_unclassified* | *Lactococcus*  *Acetobacter* |
| No.33  (7) (3B+4F) | *Tremellomycetes_unclassified, Pleosporaceae_unclassified,* | *Sphingopyxis, Ochrobactrum, Acetobacter, Aspergillus, Eurotiales_unclassified* |
| No.34  (13) (10B+3F) | *Lactobacillus, Thermomyces, Alternaria,* | *Carnobacterium, Streptococcus, Arthrobacter, Enterococcus, Acetobacter, Bacillales_unclassified, Bacillus, Lactococcus, Enhydrobacter, Aspergillus* |
| No.35  (14) (14B+0F) | *Stenotrophomonas, Pedobacter, Bacteria_unclassified, Mucilaginibacter, Luteibacter, Proteobacteria_unclassified, Aurantimonas, Weissella, Rhizobium, Sphingomonas, Comamonadaceae_unclassified, Novosphingobium, Methylobacterium, unclassified_Actinomycetales_norank,* |  |

| No.36  (16) (13B+3F) | *Acetobacter, Enhydrobacter, Halomonas, Bacillus, Lactococcus, Bacillales_unclassified, Nitriliruptor, Enterococcus, Arthrobacter, Flavobacterium, Streptococcus, Gelidibacter, Meyerozyma,* | *Lactobacillus, Thermomyces, Alternaria,* |
| --- | --- | --- |
| No.37  (21) (19B+2F) | *Staphylococcus, Luteibacter, Stenotrophomonas, Pedobacter, Roseomonas, Weissella, Rhizobium, Mucilaginibacter, Sphingomonas, Bacteria_unclassified, Xanthomonas, Sphingobacterium, Aurantimonas, Comamonadaceae_unclassified, Curtobacterium, Methylobacterium, Proteobacteria_unclassified, Novosphingobium, Fungi_unclassified,* | *Acetobacter, Aspergillus,* |
| No.38  (21) (20B+1F) | *Acetobacter, Gluconacetobacter, Enhydrobacter, Lactococcus, Sphingobium, Bacillus, Bacillales_unclassified, Brochothrix, Enterococcus, Nitriliruptor, Halomonas, Arthrobacter, Ruminococcaceae_unclassified, Streptococcus, Corynebacterium, Planococcaceae_unclassified, Aequorivita, Flavobacterium, Carnobacterium, Verticillium,* | *Lactobacillus* |
| No.39  (2) (1B+1F) |  | *Dietzia, Pseudozyma,* |
| No.40  (19) (18B+1F) | *Mucilaginibacter, Stenotrophomonas, Weissella, Aurantimonas, Pedobacter, Rhizobium, Novosphingobium, Bacteria_unclassified, Sphingomonas, Luteibacter, Roseomonas, Proteobacteria_unclassified, Staphylococcus, Methylobacterium, Xanthomonas, Sphingobacterium, Comamonadaceae_unclassified,* | *Acetobacter, Aspergillus,* |
| No.41  (5) (1B+4F) | *Phaeoseptoria, Actinomucor, Erythrobasidium, Eurotiales_unclassified,* | *Ruminococcaceae_unclassified* |
| No.42  (2) (0B+2F) | *Microdochium,* | *Fungi_unclassified,* |
| No.43  (23) (22B+1F) | *Acetobacter, Gluconacetobacter, Lactococcus, Bacillus, Enhydrobacter, Aequorivita, Bacillales_unclassified, Enterococcus, Arthrobacter, Sphingobium, Streptococcus, Corynebacterium, Ruminococcaceae_unclassified, Nitriliruptor, Carnobacterium, Planococcaceae_unclassified, Halomonas, Flavobacterium, Brochothrix, Exiguobacterium, Burkholderiales_incertae_sedis_unclassified, Verticillium,* | *Lactobacillus* |

| No.44  (24) (21B+3F) | *Acetobacter, Enhydrobacter, Lactococcus, Nitriliruptor, Gluconacetobacter, Bacillus, Bacillales_unclassified, Streptococcus, Enterococcus, Arthrobacter, Sphingobium, Planococcaceae_unclassified, Carnobacterium, Halomonas, Exiguobacterium, Ruminococcaceae_unclassified, Brochothrix, Aequorivita, Flavobacterium, Burkholderiales_incertae_sedis_unclassified, Verticillium, Aspergillus,* | *Lactobacillus, Alternaria,* |
| --- | --- | --- |
| No.45  (16) (14B+2F) | *Acetobacter, Gluconacetobacter, Enhydrobacter, Lactococcus, Nitriliruptor, Bacillus, Bacillales_unclassified, Halomonas, Enterococcus, Flavobacterium, Arthrobacter,*  *Sphingobium, ,Streptococcus, Phialosimplex,* | *Lactobacillus, Alternaria,* |
| No.46  (22) (18B+4F) | *Acetobacter, Enhydrobacter, Lactococcus, Bacillus, Gluconacetobacter, Nitriliruptor, Bacillales_unclassified, Enterococcus, Arthrobacter, Sphingobium, Streptococcus, Halomonas, Planococcaceae_u,nclassified, Carnobacterium, Flavobacterium, Aequorivita, Ruminococcaceae_unclassified, Aspergillus, Verticillium,* | *Lactobacillus, Alternaria,*  *Thermomyces,* |
| No.47  (22) (20B+2F) | *Proteobacteria_unclassified, Bacteria_unclassified, Weissella, Curtobacterium, Mucilaginibacter, Rhizobium, Staphylococcus, Comamonadaceae_unclassified, Pseudomonas, Methylobacterium, Novosphingobium, Roseomonas, Pedobacter, Luteibacter, Sphingomonas, Aurantimonas, Stenotrophomonas, Xanthomonas, Alcaligenaceae_unclassified, Sphingobacterium, Fungi_unclassified,* | *Aspergillus,* |
| No.48  (2) (1B+1F) | *Paracoccus, Unidentified,* |  |
| No.49  (16) (15B+1F) | *Acetobacter, Enhydrobacter, Lactococcus, Gluconacetobacter, Enterococcus, Halomonas, Bacillales_unclassified, Nitriliruptor, Bacillus, Arthrobacter, Sphingobium, Aequorivita, Aspergillus,* | *Sphingobacterium, Staphylococcus, Lactobacillus,* |
| No.50  (6) (3B+3F) | *Halomonas, Paracoccus, Gluconacetobacter, Unidentified, Phialosimplex,* | *Monographella,* |

| No.51  (21) (18B+3F) | *Lactobacillus, Staphylococcus, Sphingobacterium, Alternaria,* | *Nitriliruptor, Sphingobium, Halomonas, Exiguobacterium, Streptococcus, Planococcaceae_unclassified, Gluconacetobacter, Carnobacterium, Arthrobacter, Bacillus, Enterococcus, Bacillales_unclassified, Lactococcus, Enhydrobacter, Acetobacter, Verticillium, Aspergillus,* |
| --- | --- | --- |
| No.52  (6) (5B+1F) | *Acetobacter,* | *Saccharopolyspora, Roseomonas, Staphylococcus, Sphingobacterium, Fungi_unclassified,* |
| No.53  (9) (8B+1F) | *Ochrobactrum* | *Weissella, unclassified_Actinomycetales_norank, Methylobacterium, Luteibacter, Sphingobacterium, Comamonadaceae_unclassified, Staphylococcus, Fungi_unclassified,* |
| No.54  (21) (18B+3F) | *Gluconacetobacter, Ruminococcaceae_unclassified, Sphingobium, Halomonas, Lactococcus, Corynebacterium, Paracoccus, Bacillus, Bacillales_unclassified, Enterococcus, Enhydrobacter, Arthrobacter, Planococcaceae_unclassified, Flavobacterium, Streptococcus, Brochothrix, Acetobacter, Sterigmatomyces, Cryptococcus, Phialosimplex* | *Lactobacillus* |
| No.55  (12) (11B+1F) | *Gluconacetobacter, Ruminococcaceae_unclassified, Sphingobium, Corynebacterium, Lactococcus, Planococcaceae_unclassified, Bacillales_unclassified, Bacillus, Enterococcus, Halomonas, Sterigmatomyces,* | *Lactobacillus* |
| No.56  (16) (15B+1F) | *Gluconacetobacter, Ruminococcaceae_unclassified, Sphingobium, Corynebacterium, Lactococcus, Halomonas, Bacillus, Bacillales_unclassified, Enhydrobacter, Enterococcus, Planococcaceae_unclassified, Brochothrix, Arthrobacter, Streptococcus, Sterigmatomyces,* | *Lactobacillus* |
| No.57  (2) (0B+2F) | *Ascomycota_unclassified, Filobasidiaceae_unclassified,* |  |
| No.58  (26) (24B+2F) | *Lactococcus, Bacillus, Enhydrobacter, Bacillales_unclassified, Acetobacter, Arcicella, Streptococcus, Enterococcus, Gluconacetobacter, Arthrobacter, Halomonas, Leuconostoc, Exiguobacterium, Carnobacterium, Planococcaceae_unclassified, Sphingobium, Alkaliphilus, Paracoccus, Cupriavidus, Brochothrix, Flavobacterium, Propionibacterium, Ruminococcaceae_unclassified, Malassezia, Pyrenochaetopsis,* | *Lactobacillus* |

| No.59  (22) (21B+1F) | *Proteobacteria_unclassified, Weissella, Bacteria_unclassified, Rhizobium, Luteibacter, Comamonadaceae_unclassified, Methylobacterium, Novosphingobium, Mucilaginibacter, Sphingomonas, Aurantimonas, Staphylococcus, Pseudomonas, Stenotrophomonas, Pedobacter, Roseomonas, Alcaligenaceae_unclassified, Xanthomonas, Curtobacterium, Sphingobacterium, Acidovorax, Fungi_unclassified,* |  |
| --- | --- | --- |

**a-- Black font represents bacteria and green font represents fungi. *b--The character B is the abbreviation of bacteria. *c-- The character F is the abbreviation of fungi.*

**d--The yellow label represents the metabolite with weak correlation with microbiota.*

**Table S8.** The number of flavours correlated with each microbe during AAF process (|*ρ*|>0.7).

| Microbes in genus* | No. of flavours | Label of flavours |
| --- | --- | --- |
| *Acetobacter* | 56 | AA, Ala, Arg, Asp, CA, Glu, Gly, His, Ile, Leu, Lys, Met, OA, PA, PgA Pro, SA, Ser, TA, Thr, Tyr, Val,No.1, No.10, No.11, No.13, No.2, No.20, No.22, No.24, No.26, No.27, No.3, No.31, No.32, No.33, No.34 No.36 No.37 No.38 No.4, No.40, No.43, No.44, No.45, No.46, No.49, No.5, No.51, No.52, No.54 No.58, No.6, No.7, No.8 No.9, |
| *Lactobacillus* | 53 | AA, Ala, Arg, Asp, CA, Gaba, Glu, Gly, His, Ile, KgA, Leu, Lys, OA, PA, PgA, Pro, SA, Ser, TA, Thr, Val, No.1, No.10, No.11, No.13, No.17, No.21, No.22, No.24, No.25, No.26, No.27, No.28, No.3, No.34, No.36, No.38, No.4, No.43, No.44, No.45, No.46, No.49, No.5, No.51, No.54, No.55, No.56, No.58, No.6, No.7, No.8, |
| *Enhydrobacter* | 48 | AA, Ala, Arg, Asp, CA, Glu, Gly, His, Ile, KgA, Leu, Lys, OA, PA, PgA, Pro, SA, Ser, TA, Thr, Val, No.1, No.10 No.11 No.13, No.17, No.21, No.22, No.24, No.27, No.3, No.34, No.36, No.38, No.4, No.43, No.44, No.45, No.46, No.49, No.5, No.51, No.54, No.56, No.58, No.6, No.7, No.8, |
| *Lactococcus* | 46 | AA, Ala, Arg, Asp, His, KgA, Lys, OA, PA, PgA, SA, Ser, TA, Val, No.1, No.10, No.11, No.13, No.17, No.21, No.22, No.24, No.25, No.26, No.27, No.28, No.3, No.32, No.34, No.36, No.38, No.4, No.43, No.44, No.45, No.46, No.49, No.5, No.51, No.54, No.55, No.56, No.58, No.6, No.7, No.8, |
| *Bacillales_unclassified* | 44 | Fruc, AA, Ala, Arg, Asp, His, KgA, Lys, OA, PA, PgA, SA, Ser, TA, Val, No.1, No.10, No.11, No.13, No.17, No.21, No.22, No.24, No.27, No.28, No.3, No.34, No.36, No.38, No.4, No.43, No.44, No.45, No.46, No.49, No.5, No.51, No.54, No.55, No.56, No.58, No.6, No.7, No.8, |

| *Bacillus* | 42 | AA, Ala, Arg, ,Asp, His, KgA, Lys, OA, PA, PgA, SA, Ser, TA,No.1, No.10, No.11, No.13, No.17, No.21, No.22, No.24, No.27, No.28, No.3, No.34, No.36, No.38, No.4, No.43, No.44, No.45, No.46, No.49, No.5, No.51, No.54, No.55, No.56, No.58, No.6, No.7, No.8, |
| --- | --- | --- |
| *Gluconacetobacter* | 42 | AA, Arg, CA, His, KgA, Lys, OA, PA, PgA, SA, Ser, TA, No.1, No.10, No.11, No.13, No.17, No.21, No.22, No.24, No.25, No.26, No.27, No.28, No.3, No.38, No.4, No.43, No.44, No.45, No.46, No.49, No.5, No.50, No.51, No.54, No.55, No.56, No.58, No.6, No.7, No.8, |
| *Enterococcus* | 41 | AA, Ala, Arg, Asp, His, KgA, Lys, OA, PA, PgA, SA, Ser, TA, No.1, No.10, No.11, No.13, No.17, No.21, No.22, No.24, No.28, No.3, No.34, No.36, No.38, No.4, No.43, No.44, No.45, No.46, No.49, No.5, No.51, No.54, No.55, No.56, No.58, No.6, No.7, No.8, |
| *Arthrobacter* | 39 | AA, Ala, Arg, Asp, His, Lys, OA, PA, PgA, SA, Ser, TA, No.1, No.10, No.11, No.13, No.17, No.21, No.22, No.24, No.28, No.3, No.34, No.36, No.38, No.4, No.43, No.44, No.45, No.46, No.49, No.5, No.51, No.54, No.56, No.58, No.6, No.7, No.8, |
| *Aspergillus* | 39 | AA, Ala, Arg, Asp, Cys, Glu, Gly, Ile, Leu, Lys, Met, OA, PgA, Phe, Pro, Thr, Tyr, Val, No.1, No.10, No.11, No.12, No.18, No.2, No.20, No.3, No.31, No.33, No.34, No.37, No.4, No.40, No.44, No.46, No.47, No.49, No.51, No.7, No.9, |
| *Staphylococcus* | 41 | Fruc, Gluc, Ala, Asp, CA, Cys, Glu, Gly, Ile, LA, Leu, Met, OA, Phe, Pro, Thr, Tyr, Val, No.1, No.12, No.14, No.15, No.16, No.19, No.2, No.20, No.21, No.22, No.23, No.3, No.31, No.32, No.37, No.4, No.40, No.47, No.49, No.51, No.52, No.53, No.59, |
| *Sphingobium* | 37 | AA, Arg, His, KgA, Lys, PA, PgA, SA, Ser, TA, No.1, No.10, No.11, No.13, No.21, No.22, No.24, No.25, No.26, No.27, No.28, No.38, No.4, No.43, No.44, No.45, No.46, No.49, No.5, No.51, No.54, No.55, No.56, No.58, No.6, No.7, No.8, |
| *Sphingobacterium* | 36 | Fruc, Ala, Asp, CA, Cys, Glu, Gly, Ile, LA, Leu, Met, OA, Phe, Pro, Thr, Tyr, Val, No.12, No.14, No.15, No.16, No.19, No.2, No.21, No.22, No.3, No.31, No.32, No.37, No.40, No.47, No.49, No.51, No.52, No.53, No.59, |
| *Streptococcus* | 34 | AA, Arg, Gaba, ,His, KgA, Lys, PA, PgA, SA, Ser, TA, No.1, No.10, No.11, No.13, No.17, No.24, No.28, No.34, No.36, No.38, No.4, No.43, No.44, No.45, No.46, No.5, No.51, No.54, No.56 , No.58, No.6, No.7, No.8, |
| *Planococcaceae_unclassified* | 33 | AA, Arg, His, KgA, Lys, PA, PgA, SA, Ser, TA, No.1, No.10, No.11, No.13, No.17, No.21, No.22, No.24, No.28, No.38, No.4, No.43, No.44, No.46, No.5, No.51, No.54, No.55, No.56, No.58, No.6, No.7, No.8, |
| *Fungi_unclassified* | 34 | Fruc, Gluc, Ala, Asp, Cys, Glu, Gly, Ile, LA, Leu, Met, OA, Phe, Pro, Thr, Tyr, Val, No.12, No.14, No.15, No.16, No.2, No.20, No.22, No.23, No.3, No.31, No.32, No.37, No.42, No.47, No.52, No.53, No.59, |

| *Halomonas* | 32 | AA, Arg, His, Lys, PA, PgA, SA, Ser, TA, No.1, No.10, No.11, No.13, No.24, No.27, No.36, No.38, No.43, No.44, No.45, No.46, No.49, No.5, No.50, No.51, No.54, No.55, No.56, No.58, No.6, No.7, No.8, |
| --- | --- | --- |
| *Roseomonas* | 33 | Fruc, Ala, Asp, CA, Cys, Glu, Gly, Ile, LA, Leu, Met, OA, Phe, Pro, Thr, Tyr, Val, No.12, No.14, No.15, No.16, No.2, No.20, No.21, No.22, No.3, No.31, No.32, No.37, No.40, No.47, No.52, No.59, |
| *Ruminococcaceae_unclassified* | 32 | AA, Arg, His, KgA, Lys, PA, PgA, SA, Ser, TA, No.10, No.11, No.13, No.21, No.22, No.24, No.25, No.27, No.28, No.38, No.41, No.43, No.44, No.46, No.5, No.54, ,No.55, No.56, No.58, No.6, No.7, No.8, |
| *Carnobacterium* | 30 | AA, Arg, His, Lys, PA, PgA, SA, Ser, No.1, No.10, No.11, No.13, No.17, No.21, No.22, No.24, No.28, No.3, No.34, No.38, No.4, No.43, No.44, No.46, No.5, No.51, No.58, No.6, No.7, No.8, |
| *Luteibacter* | 31 | Fruc, Ala, Cys, Glu, Gly, Ile, LA, Leu, Met, OA, Phe, Pro, Thr, Tyr, Val, No.12, No.14, No.15, No.16, No.19, No.2, No.22, No.3, No.31, No.32, No.35, No.37, No.40, No.47, No.53, No.59, |
| *Verticillium* | 30 | AA, Arg, Asp, His, Lys, OA, PA, PgA, SA, Ser, TA, No.1, No.10, No.11, No.13, No.21, No.22, No.24, No.25, No.3, No.38, No.4, No.43, No.44, No.46, No.5, No.51, No.6, No.7, No.8, |
| *Stenotrophomonas* | 29 | Fruc, Ala, Asp, Cys, Glu, Gly, Ile, Leu, Met, OA, Phe, Pro, Thr, Tyr, Val, No.12, No.14, No.15, No.16, No.2, No.22, No.3, No.31, No.32, No.35, No.37, No.40, No.47, No.59, |
| *Weissella* | 30 | Fruc, Gluc, Cys, Glu, Gly, Ile, LA, Leu, Met, Phe, Pro, Thr, Tyr, Val, No.12, No.14, No.15, No.16, No.19, No.2, No.22, No.23, No.31, No.32, No.35, No.37, No.40, No.47, No.53, No.59, |
| *Brochothrix* | 27 | AA, Arg, His, Lys, PA, PgA, SA, Ser, TA, No.1, No.10, No.11, No.13, No.21, No.22, No.24, No.28, No.38, No.43, No.44, No.5, No.54, No.56, No.58, No.6, No.7, No.8, |
| *Nitriliruptor* | 27 | AA, Arg, Lys, PA, PgA, SA, TA, No.1, No.11, No.13, No.24, No.26, No.27, No.3, No.36, No.38, No.4, No.43, No.44, No.45, No.46, No.49, No.5, No.51, No.6, No.7, No.8, |
| *Bacteria_unclassified* | 25 | Cys, Glu, Gly, Ile, LA, Leu, Met, Phe, Pro, Thr, No.12, No.14, No.15, No.16, No.19, No.2, No.22, No.23, No.31, No.32, No.35, No.37, No.40, No.47, No.59, |
| *Comamonadaceae_unclassified* | 24 | Fruc, Cys, Gly, LA, Leu, Met, Phe, Pro, Thr, No.12, No.14, No.15, No.16, No.2, No.20, No.23, No.31, No.32, No.35, No.37, No.40, No.47, No.53, No.59, |

| *Exiguobacterium* | 23 | AA, Arg, His, Lys, PA, PgA, SA, Ser, No.1, No.10, No.11, No.13, No.17, No.24, No.28, No.43, No.44, No.5, No.51, No.58, No.6, No.7, No.8, |
| --- | --- | --- |
| *Methylobacterium* | 23 | Fruc, Cys, Gly, LA, Leu, Phe, Thr, No.12, No.14, No.15, No.16, ,No.19, No.2, No.22, No.23, No.31, No.32, No.35, No.37, No.40, No.47, No.53, No.59, |
| *Rhizobium* | 23 | Fruc, Cys, Glu, Gly, LA, Leu, Phe, Thr, No.12, No.14, No.15, No.16, No.19, No.2, No.22, No.23, No.31, No.32, No.35, No.37, No.40, No.47, No.59, |
| *Proteobacteria_unclassified* | 22 | Fruc, Cys, Gly, LA, Leu, Met, Phe, Thr, No.12, No.14, No.15, No.16, No.19, No.2, No.23, No.31, No.32, No.35, No.37, No.40, No.47, No.59, |
| *Flavobacterium* | 20 | AA, Arg, His, SA, Ser, No.10, No.11, No.13, No.24, No.36, No.38, No.43, No.44, No.45, No.46, No.5, No.54, No.58, No.6, No.8, |
| *Aurantimonas* | 20 | Fruc, Cys, Gly, Leu, Phe, Thr, No.12, No.14, No.15, No.16, No.19, No.2, No.22 No.31, No.32, No.35, No.37, No.40, No.47, No.59, |
| *Mucilaginibacter* | 20 | Fruc, Cys, Gly, Leu, Phe, Pro, Thr, No.12, No.14, No.15, No.16, No.2, No.22, No.31, No.32, No.35, No.37, No.40, No.47, No.59, |
| *Sphingomonas* | 20 | Fruc, Cys, Gly, Leu, Phe, Thr, No.12, No.14 No.15, No.16, No.19, No.2, No.22, No.31, No.32, No.35, No.37, No.40, No.47, No.59, |
| *Alternaria* | 17 | AA, Arg, Gaba, Lys, PgA, ,TA, No.1, No.17, No.26, No.34, No.36, No.44, No.45, No.46, No.5, No.51, No.7, |
| *Novosphingobium* | 18 | Fruc, Cys, LA, Phe, No.12, No.14, No.15, No.16, No.19, No.2, No.23, No.31, No.32, No.35, No.37, No.40, No.47, No.59, |
| *Pedobacter* | 18 | Fruc, Cys, Leu, Phe, Thr, No.12, No.14, No.15, No.16, No.2, No.22, No.31, No.32, No.35, No.37, No.40, No.47, No.59, |
| *Xanthomonas* | 18 | Fruc, Cys, Leu, Phe, No.12, No.14, No.15, No.16, No.2, No.21, No.22, No.25, No.31, No.32, No.37, No.40, No.47, No.59, |
| *Aequorivita* | 16 | AA, Arg, ,Lys, PA, PgA, SA, No.13, No.24, No.38, No.43, No.44, No.46, No.49, No.5, No.6, No.7, |
| *Corynebacterium* | 16 | PA, PgA, SA, Ser, TA, No.13, No.24, No.25, No.27, No.38, No.43, No.54, No.55, No.56, No.6, No.7, |
| *Eurotiales_unclassified* | 16 | Ala, Asp, Cys, Glu, Gly, Ile, Leu, Met, Phe, Thr, Tyr, Val, No.20, No.33, No.41, No.9, |
| *Burkholderiales_incertae_sedis_unclassified* | 15 | AA, Arg, His, PA, PgA, SA, Ser, No.1, No.11, No.13, No.24, No.28, No.43, No.44, No.5, |
| *Curtobacterium* | 14 | Fruc, Cys, No.12, No.14, No.15, No.16, No.2, No.21, No.22, No.31, No.32, No.37, No.47, No.59, |
| *Pseudomonas* | 13 | Fruc, LA, No.12, No.14, No.15, No.16, No.19, No.2, No.23, No.29, No.31, No.47, No.59, |
| *Malassezia* | 10 | AA, Arg, His, Lys, Ser, No.13, No.24, No.28, No.5, No.58, |
| *Paracoccus* | 9 | Ser, No.24, No.27, No.48, No.50, No.54, No.58, No.6, No.8, |

| *Acidovorax* | 8 | No.12, No.14, No.15, No.16, No.19, No.23, No.29, No.59, |
| --- | --- | --- |
| *Alcaligenaceae_unclassified* | 8 | No.12, No.14, No.15, No.16, No.2, No.31, No.47, No.59, |
| *Alkaliphilus* | 7 | Arg, His, Ser, No.13, No.24, No.5, No.58, |
| *Leuconostoc* | 7 | His, Ser, No.13, No.17, No.5, No.58, No.8, |
| *Sterigmatomyces* | 7 | PA, SA, No.24, No.27, No.54, No.55, No.56, |
| *Pleosporales_unclassified* | 6 | KgA, No.19, No.23, No.26, No.29, No.5, |
| *Massilia* | 5 | Gaba, LA, No.23, No.29, No.30, |
| *Sarocladium* | 5 | KgA, No.23, No.26, No.28, No.29, |
| *Thermomyces* | 5 | Gaba, Lys, No.34, No.36, No.46, |
| *Kineococcus* | 4 | Cys, No.21, No.31, No.32, |
| *Phialosimplex* | 4 | No.27, No.45, No.50, No.54, |
| *Dietzia* | 3 | His, No.39, Ser, |
| *Fusarium* | 3 | LA, No.23, No.28, |
| *Pseudozyma* | 3 | His, No.25, No.39, |
| *Sphingopyxis* | 3 | No.18, No.20, No.33, |
| *unclassified_Actinomycetales_norank* | 3 | No.20, No.35, No.53, |
| *Aquabacterium* | 2 | No.23, No.29, |
| *Arcicella* | 2 | No.58, No.7, |
| *Cryptococcus* | 2 | No.27, No.54, |
| *Cystobasidiomycetes_unclassified* | 2 | KgA, No.23, |
| *Epicoccum* | 2 | Gaba, No.5, |
| *Gelidibacter* | 2 | No.11, No.36, |
| *Meyerozyma* | 2 | No.36, No.8, |
| *Ochrobactrum* | 2 | No.33, No.53, |

| *Phaeoseptoria* | 2 | LA, No.41, |
| --- | --- | --- |
| *Propionibacterium* | 2 | His, No.58, |
| *Saccharopolyspora* | 2 | No.32, No.52, |
| *Thermomonas* | 2 | No.5, Ser, |
| *unidentified* | 2 | No.48, No.50, |
| *Wautersiella* | 2 | No.21, No.32, |
| *Actinomucor* | 1 | No.41 |
| *Ascomycota_unclassified* | 1 | No.57 |
| *Cupriavidus* | 1 | No.58 |
| *Erythrobasidium* | 1 | No.41 |
| *Filobasidiaceae_unclassified* | 1 | No.57 |
| *Leptosphaeria* | 1 | No.23 |
| *Microdochium* | 1 | No.42 |
| *Monographella* | 1 | No.50 |
| *Mycosphaerellaceae_unclassified* | 1 | Gaba |
| *Phaeoacremonium* | 1 | Ser |
| *Pleosporaceae_unclassified* | 1 | No.33 |
| *Pyrenochaetopsis* | 1 | No.58 |
| *Rhizomucor* | 1 | No.19 |
| *Tremellomycetes_unclassified* | 1 | No.33 |
| *Trichosphaeriales_unclassified* | 1 | No.23 |
| *Veillonella* | 1 | No.25 |

**Black font represents bacteria and green font represents fungi.*

**Table S9.** The number of flavours highly correlated (|*ρ*|>0.8) with each microbe during AAF process.

| Microbes in genus* | No. of flavours | Label of flavours |
| --- | --- | --- |
| Acetobacter | 41(7OAs+12AAs+22VFs) | OA, TA, PA, AA, CA, PgA, SA, Pro, Asp, Glu, His, Gly, Thr, Arg, Ala, Val, Ile, Leu, Lys, No.44, No.7, No.11, No.43, No.46, No.13, No.6, No.36, No.10, No.24, No.38, No.8, No.45, No.49, No.58, No.26, No.34, No.5, No.3, No.4, No.51, No.1, |
| Gluconacetobacter | 35(7OAs+4AAs+24VFs) | TA, PA, KgA, AA, CA, PgA, SA, Lys, Ser, His, Arg, No.56, No.55, No.24, No.54, No.13, No.43, No.8, No.27, No.6, No.44, No.11, No.7, No.38, No.58, No.45, No.46, No.10, No.28, No.17, No.26, No.22, No.21, No.5, No.25, |
| Lactobacillus | 34(7OAs+5AAs+22VFs) | OA, TA, PA, AA, CA, PgA, SA, Lys, Asp, His, Arg, Ala, No.5, No.1, No.34, No.51, No.4, No.3, No.26, No.17, No.49, No.38, No.10, No.45, No.36, No.58, No.6, No.46, No.8, No.13, No.24, No.11, No.7, No.44, |
| Enhydrobacter | 32(6OAs+5AAs+21VFs) | OA, PA, AA, CA, PgA, SA, Asp, Ser, His, Arg, Lys, No.13, No.58, No.7, No.44, No.24, No.6, No.43, No.10, No.8, No.11, No.46, No.38, No.49, No.45, No.17, No.34, No.3, No.4, No.51, No.1, No.5, |
| Staphylococcus | 28(3OAs+11AAs+14VFs) | OA, LA, CA, Pro, Glu, Gly, Thr, Ala, Tyr, Val, Met, Phe, Ile, Leu, No.31, No.2, No.59, No.16, No.12, No.37, No.15, No.14, No.47, No.32, No.22, No.19, No.53, No.52, |
| Lactococcus | 27(4OAs+4AAs+19VFs) | PA, AA, PgA, SA, Lys, Ser, His, Arg, No.13, No.58, No.24, No.43, No.44, No.8, No.6, No.7, No.11, No.10, No.38, No.46, No.28, No.26, No.17, No.4, No.51, No.1, No.5, |
| Bacillus | 26(5OAs+4AAs+17VFs) | TA, PA, AA, PgA, SA, Lys, Ser, His, Arg, No.58, No.13, No.24, No.8, No.43, No.44, No.7, No.6, No.11, No.10, No.46, No.38, No.28, No.17, No.51, No.1, No.5, |
| Bacillales_unclassified | 25(4OAs+4AAs+17VFs) | PA, AA, PgA, SA, Lys, Ser, His, Arg, No.59, No.15, No.14, No.16, No.12, No.31, No.47, No.2, No.37, No.35, No.19, |
| Enterococcus | 23(4OAs+4AAs+15VFs) | PA, AA, PgA, SA, Lys, Ser, His, Arg, No.13, No.58, No.24, No.43, No.44, No.8, No.6, No.10, No.7, No.11, No.28, No.17, No.51, No.1, No.5, |
| Sphingobacterium | 20(2OAs+9AAs+9VFs) | LA, CA, Leu, Glu, Gly, Thr, Tyr, Val, Met, Phe, Ile, No.31, No.2, No.37, No.32, No.59, No.16, No.12, No.19, No.52, |
| Arthrobacter | 19(3OAs+4AAs+12VFs) | AA, PgA, SA, Ser, His, Arg, Lys, No.13, No.58, No.24, No.43, No.44, No.8, No.6, No.10, No.11, No.17, No.1, No.5, |
| Sphingobium | 18(3OAs++13VFs) | PA, AA, SA, Lys, Arg, No.56, No.55, No.24, No.6, No.13, No.43, No.8, No.54, No.44, No.26, No.5, No.21, No.25, |
| Roseomonas | 16(1OAs+6AAs+9VFs) | CA, Leu, Glu, Gly, Thr, Cys, Phe, No.31, No.12, No.59, No.16, No.37, No.2, No.15, No.32, No.14, |
| Streptococcus | 15(2OAs+4AAs+9VFs) | AA, PgA, Lys, Ser, His, Arg, No.58, No.13, No.24, No.8, No.43, No.44, No.28, No.17, No.5, |
| Stenotrophomonas | 14(1AAs+13VFs) | Phe, No.12, No.31, No.59, No.16, No.14, No.15, No.2, No.37, No.47, No.35, No.32, No.40, No.22, |

| Luteibacter | 13(2AAs+11VFs) | Phe, Cys, No.12, No.59, No.16, No.31, No.15, No.14, No.2, No.37, No.47, No.32, No.19, |
| --- | --- | --- |
| Weissella | 13(1OAs+1AAs+11VFs) | LA, Phe, No.59, No.15, No.16, No.12, No.14, No.31, No.47, No.2, No.37, No.40, No.19, |
| Aurantimonas | 12(1AAs+11VFs) | Cys, No.12, No.59, No.16, No.14, No.15, No.31, No.2, No.47, No.37, No.40, No.19, |
| Bacteria_unclassified | 12(1AAs+11VFs) | Phe, No.59, No.15, No.14, No.16, No.12, No.31, No.47, No.2, No.37, No.35, No.19, |
| Pedobacter | 12(2AAs+10VFs) | Phe, Cys, No.12, No.59, No.15, No.16, No.14, No.31, No.2, No.35, No.40, No.37, |
| Proteobacteria_unclassified | 12(1OAs+1AAs+10VFs) | LA, Phe, No.15, No.59, No.14, No.16, No.12, No.47, No.31, No.2, No.37, No.19, |
| Rhizobium | 12(1AAs+11VFs) | Phe, No.12, No.59, No.16, No.14, No.15, No.31, No.47, No.2, No.37, No.40, No.19, |
| Ruminococcaceae_unclassified | 12(2OAs+1AAs+9VFs) | PA, SA, Ser, No.55, No.56, No.54, No.24, No.13, No.43, No.28, No.21, No.25, |
| Mucilaginibacter | 11(11VFs) | No.12, No.59, No.14, No.16, No.15, No.31, No.47, No.40, No.37, No.2, No.32, |
| Planococcaceae_unclassified | 11(3OAs+3AAs+5VFs) | PA, AA, SA, Arg, Ser, His, No.13, No.24, No.11, No.17, No.5, |
| Sphingomonas | 11(11VFs) | No.12, No.59, No.16, No.14, No.15, No.31, No.2, No.47, No.37, No.32, No.19, |
| Methylobacterium | 10(10VFs) | No.59, No.12, No.16, No.15, No.14, No.31, No.2, No.47, No.37, No.19, |
| Novosphingobium | 10(10VFs) | No.59, No.12, No.14, No.15, No.16, No.31, No.47, No.2, No.37, No.19, |
| Comamonadaceae_unclassified | 9(9VFs) | No.59, No.12, No.16, No.15, No.14, No.31, No.47, No.2, No.37, |
| Halomonas | 9(2AAs+7VFs) | His, Ser, No.58, No.7, No.6, No.24, No.13, No.10, No.50, |
| Xanthomonas | 9(9VFs) | No.12, No.31, No.59, No.16, No.2, No.14, No.32, No.37, No.15, |
| Carnobacterium | 8(1OAs+3AAs+4VFs) | AA, Ser, His, Arg, No.13, No.58, No.17, No.5, |
| Pseudomonas | 8(1OAs+7VFs) | LA, No.15, No.14, No.59, No.16, No.12, No.47, No.19, |
| Alcaligenaceae_unclassified | 5(5VFs) | No.59, No.12, No.15, No.14, No.16, |
| Curtobacterium | 5(5VFs) | No.12, No.31, No.59, No.2, No.16, |
| Exiguobacterium | 4(2AAs+2VFs) | His, Ser, No.58, No.17, |
| Brochothrix | 3(2AAs+1VFs) | His, Ser, No.13, |
| Nitriliruptor | 3(3VFs) | No.44, No.43, No.26, |
| Leuconostoc | 2(1AAs+1VFs) | Ser, No.17 |
| Acidovorax | 1(1VFs) | No.19 |

| Alkaliphilus | 1(1AAs) | His |
| --- | --- | --- |
| Burkholderiales_incertae_sedis_unclassified | 1(1AAs) | His |
| Corynebacterium | 1(1VFs) | No.56 |
| Massilia | 1(1VFs) | No.30 |
| Paracoccus | 1(1VFs) | No.48 |
| Sphingopyxis | 1(1VFs) | No.18 |
| Aspergillus | 17(1OAs+10AAs+6VFs) | OA, Leu, Asp, Glu, Gly, Thr, Ala, Tyr, Val, Met, Ile, No.20, No.9, No.1, No.3, No.4, No.51, |
| Fungi_unclassified | 17(1OAs+7AAs+9VFs) | LA, Leu, Glu, Gly, Thr, Met, Phe, Ile, No.2, No.31, No.59, No.16, No.12, No.15, No.37, No.14, No.47, |
| Verticillium | 5(2OAs +3AAs) | PgA, SA, Arg, Ser, His, |
| Alternaria | 4(1AAs+3VFs) | Arg, No.5, No.17, No.26, |
| Eurotiales_unclassified | 4(1AAs+3VFs) | Met, No.20, No.33, No.9, |
| Pleosporales_unclassified | 3(3VFs) | No.23, No.29, No.26, |
| Sarocladium | 3(3VFs) | No.23, No.29, No.26, |
| Actinomucor | 1(1VFs) | No.41 |
| Ascomycota_unclassified | 1(1VFs) | No.57 |
| Epicoccum | 1(1AAs) | Gaba |
| Erythrobasidium | 1(1VFs) | No.41 |
| Filobasidiaceae_unclassified | 1(1VFs) | No.57 |
| Microdochium | 1(1VFs) | No.42 |
| Phaeoseptoria | 1(1VFs) | No.41 |
| Phialosimplex | 1(1VFs) | No.27 |
| Pleosporaceae_unclassified | 1(1VFs) | No.33 |
| Pseudozyma | 1(1VFs) | No.39 |
| Thermomyces | 1(1VFs) | No.34 |
| Tremellomycetes_unclassified | 1(1VFs) | No.33 |
| unidentified | 1(1VFs) | No.48 |

**Black font represents bacteria and green font represents fungi.*

**Table S10.** Detailed information of the functional core microbiota in vinegar *Pei* during AAF process.

| Highly correlated flavours (|*ρ*|>0.8) | | *Acetobacter*  **(G1)** | *Lactobacillus*  **(G2)** | *Enhydrobacter*  **(G3)** | *Lactococcus*  **(G4)** | *Gluconacetobacter* **(G6)** | *Bacillus*  **(G7)** | *Staphylococcus*  **(G10)** |
| --- | --- | --- | --- | --- | --- | --- | --- | --- |
| *VIP_(pred)_* | | 1.628 | 1.628 | 1.598 | 1.602 | 1.606 | 1.571 | 1.576 |
| Organic acids (9) | | OA, TA, PA, AA,  CA, PgA, SA | OA, TA, PA, AA,  CA, PgA, SA | OA, PA, AA,  CA, PgA, SA | PA, AA,  PgA, SA | TA, PA, KgA, AA, CA, PgA, SA | TA, PA, AA,  PgA, SA | OA, LA, CA |
| Amino acids (16) | | Pro, Asp, Glu, His, Gly, Thr, Arg, Ala, Val, Ile, Leu, Lys | Lys, Asp, His,  Arg, Ala | Asp, Ser, His,  Arg, Lys | Lys, Ser,  His, Arg | Lys, Ser,  His, Arg | Lys, Ser,  His, Arg | Pro, Glu, Gly, Thr, Ala, Tyr, Val, Met, Phe, Ile, Leu |
| Volatile flavours  (44) | Alcohols | No.1, No.3, No.4, No.5, No.6, No.7, No.8 | No.1, No.3, No.4, No.5, No.6, No.7, No.8 | No.1, No.3, No.4, No.5, No.6, No.7, No.8 | No.1, No.4, No.5, No.6, No.7, No.8 | No.5, No.6,  No.7, No.8, | No.1, No.5, No.6, No.7, No.8 | No.2 |
|  | Acids | No.10, No.11,  No.13 | No.10, No.11,  No.13, No.17 | No.10, No.11,  No.13, No.17 | No.11, No.10, No.13, No.17 | No.10, No.11,  No.13, No.17 | No.10, No.11, No.13, No.17 | No.12, No.14,  No.15, No.16 |
|  | Esters | No.24, No.26, No.34,  No.36, No.38 | No.24, No.26, No.34, No.36, No.38 | No.24, No.34,  No.38 | No.24, No.26, No.28, No.38 | No.21, No.22, No.24, No.25, No.26, No.27, No.28, No.38 | No.24, No.28, No.38 | No.19, No.22, No.31,  No.32, No.37 |
|  | Ketones | No.43, No.44,  No.45, No.46 | No.44, No.45,  No.46 | No.43, No.44,  No.45, No.46 | No.43, No.44, No.46, | No.43, No.44,  No.45, No.46 | No.43, No.44, No.46 | -- |
|  | Aldehydes | No.49, No.51 | No.49, No.51 | No.49, No.51 | No.51 | -- | No.51 | No.47, No.52, No.53 |
|  | Heterocycles | -- | -- | -- | -- | No.54, No.55, No.56 | -- | -- |
|  | Others | No.58 | No.58, | No.58 | No.58 | No.58 | No.58 | No.59 |

**Table S11.** Comparsion of flavours between control batch and bioaugmentation of *A. pasteurianus*.

| Flavours | | | Control | Bioaugmentation of *A. pasteurianus** | | O2PLS  (coefficient, *ρ*) |
| --- | --- | --- | --- | --- | --- | --- |
| Organic acids (g/100 g dry culture) | PgA | Pyroglutamic acid | 0.73 | 0.86 | 0.95 | |
|  | AA | Acetic acid | 9.32 | 10.45 | 0.94 | |
|  | SA | Succinic acid | 1.13 | 1.66 | 0.88 | |
|  | TA | Tartaric acid | 0.49 | 0.04 | 0.81 | |
|  | CA | Citric acid | 1.31 | 0.48 | 0.79 | |
|  | KgA | Ketoglutaric acid | 0.00 | 0.57 | 0.57 | |
|  | LA | Lactic acid | 2.60 | 2.67 | 0.04 | |
| Amino acids (mg/100 g dry culture) | Arg | Arginine | 293.11 | 298.72 | 0.94 | |
|  | Lys | Lysine | 257.64 | 243.30 | 0.90 | |
|  | Ala | Alanine | 467.41 | 487.29 | 0.89 | |
|  | Asp | Aspartic acid | 275.94 | 276.91 | 0.88 | |
|  | Val | Valine | 309.23 | 251.21 | 0.86 | |
|  | His | Histidine | 9.39 | 7.51 | 0.85 | |
|  | Glu | Glutamic acid | 608.62 | 619.34 | 0.83 | |
|  | Ile | Isoleucine | 175.72 | 188.84 | 0.83 | |
|  | Thr | Threonine | 121.55 | 127.18 | 0.83 | |
|  | Pro | Proline | 161.09 | 174.13 | 0.82 | |
|  | Gly | Glycine | 179.37 | 181.82 | 0.82 | |
|  | Leu | Leucine | 422.75 | 437.27 | 0.80 | |
|  | Tyr | Tyrosine | 112.58 | 121.33 | 0.78 | |
|  | Ser | Serine | 50.10 | 65.14 | 0.71 | |
|  | Met | Methionine | 119.06 | 121.24 | 0.71 | |
|  | Phe | Phenylalanine | 165.51 | 145.18 | 0.62 | |
|  | Gaba | γ-aminobutyric acid | 42.54 | 37.97 | 0.58 | |
|  | Cys | Cysteine | 17.23 | 14.73 | 0.53 | |
| Volatile (alcohols)  μg/100 g dry culture | No.6 | 2,3-butanediol | 0.00 | 9.44 | 0.90 | |
|  | No.1 | alcohol | 0.00 | 4.83 | -0.95 | |
|  | No.2 | 2-methyl-1-propanol | 7.37 | 3.93 | -0.66 | |
|  | No.3 | 3-methyl-1-butanol | 50.97 | 43.65 | -0.90 | |
|  | No.9 | Phenylethyl alcohol | 80.67 | 63.59 | -0.64 | |
| Volatile (acids)  μg/100 g dry culture | No.15 | Heptanoic acid | 2.01 | 2.13 | -0.33 | |
|  | No.11 | 2-methyl-propanoic acid | 16.49 | 11.68 | 0.92 | |
|  | No.14 | Hexanoic acid | 14.99 | 12.49 | -0.32 | |
|  | No.16 | Octanoic acid | 9.79 | 8.22 | -0.42 | |
| Volatile (esters)  μg/100 g dry culture | No.36 | Ethyl phenylacetate | 3.98 | 4.06 | 0.90 | |
|  | No.20 | Isopentyl acetate | 22.52 | 9.95 | 0.64 | |
|  | No.18 | Ethyl acetate | 222.48 | 81.42 | 0.36 | |
|  | No.41 | Ethyl palmitate | 116.96 | 80.81 | 0.21 | |
|  | No.42 | Ethyl oleate | 33.87 | 24.52 | -0.01 | |
|  | No.39 | Ethyl laurate | 5.46 | 4.07 | -0.19 | |
|  | No.29 | Isoamyl lactate | 11.85 | 8.48 | -0.37 | |
|  | No.28 | 2-hydroxy-4-methyl-ethyl valerate | 54.26 | 27.32 | -0.47 | |
|  | No.25 | Ethyl caprylate | 5.11 | 3.88 | -0.51 | |
|  | No.32 | Ethyl caprate | 8.32 | 4.80 | -0.69 | |
|  | No.22 | Ethyl caproate | 1.13 | 1.07 | -0.79 | |
|  | No.34 | Diethyl succinate | 64.16 | 48.15 | -0.89 | |

| Volatile (ketones)  μg/100 g dry culture | No.43 | 2,3-butanedione | 0.00 | 0.97 | 0.91 |
| --- | --- | --- | --- | --- | --- |
|  | No.46 | 5-heptyl dihydro-2(3H)-Furanone | 3.68 | 3.91 | 0.91 |
|  | No.44 | 3-hydroxy-2-butanone | 61.93 | 37.52 | 0.95 |
| Volatile (aldehydes)  μg/100 g dry culture | No.52 | alpha-ethylidene-phenylacetaldehyde | 2.62 | 1.91 | 0.70 |
|  | No.50 | benzaldehyde | 4.62 | 3.36 | 0.51 |
| Volatile (others)  μg/100 g dry culture | No.58 | 2-methoxy-4-methyl-phenol | 2.72 | 13.41 | 0.87 |
|  | No.56 | 2,3,5,6-tetramethyl-pyrazine | 4.02 | 7.45 | 0.69 |
|  | No.57 | methoxy-phenyl-oxime | 0.00 | 8.37 | -0.14 |

********Red colour represents the level of flavour is increasing while green colour represents the level of flavour is decreasing.*

**Table S12.** Primers used for Miseq and biomass analysis in this study.

| Primers | Sequences used in this study |
| --- | --- |
| Miseq primers of bacteria | 515F (5’-GTGCCAGCMGCCGCGG-3’) |
|  | 907R (5’-CCGTCAATTCMTTTRAGTTT-3’) |
| Miseq primers of fungi | 1737F (5’-GGAAGTAAAAGTCGTAACAAGG-3’) |
|  | 2043R (5’-GCTGCGTTCTTCATCGATGC-3’) |
| Biomass primers of bacteria | 340F (5’-CCTACGGGAGGCAGCAG-3’) |
|  | 758R (5’-CTACCAGGGTATCTAATCC-3’) |
| Biomass primers of fungi | Y1 (5’-GCGGTAATTCCAGCTCCAATAG-3’) |
|  | Y2 (5’-GCCACAAGGACTCAAGGTTAG-3’) |

**Supplementary methods**

**Extraction of metagenomic DNA from vinegar *Pei***

Wet vinegar *Pei* (about 3.5 g, moisture content≈60%) was mixed with 13.5 mL of DNA extraction buffer (100 mM Tris-HCl, 100 mM sodium EDTA, 100 mM sodium phosphate, 1.5 M NaCl, 1% CTAB, pH 8.0) and 80 µL of proteinase K (10 mg/mL) in centrifuge tube with horizontal shaking. Then, 2 mL SDS (10%) was added, and the samples were incubated in water bath (65^○^C) for 3 h. After centrifugation the supernatants of extraction were mixed with an equal volume of chloroform-isoamyl alcohol (24:1, v/v). The aqueous phase was recovered by centrifugation and precipitated with 0.6 volumes of isopropanol. A pellet of crude nucleic acids was obtained by centrifugation, washed with pre-chilled ethanol (70%) and resuspended in sterile TE buffer to give a final volume of 200 µL. Concentrations of total DNA were measured using a DyNA quant 200(Hoefer, San Francisco, CA, USA). DNA purity was determined by A260/A280. DNA integrity was verified by 1% agarose gel electrophoresis.

**PCR-amplification and sequencing**

The primers of bacteria and fungi were incorporated with a unique 6-8 nt as barcode to discriminate each sample. All primer barcodes used are presented in Dataset S1. For bacteria, PCR reactions contain 10 ng DNA templates, 0.4 μL FastPfu Polymerase (TransGen Biotech, Beijing, China), 4 μL FastPfu Buffer (5×), 2 μL dNTPs (2.5 mM), 0.8 μL each primer (5 μM) and adding ddH_2_O to 20 μL. Reaction conditions consisted of an initial 95°C for 3 min followed by 27 cycles of 95°C for 30 s, 55°C for 30 s, and 72°C for 45 s, and a final extension of 72°C for 10 min. For fungi, PCR reactions was the same as bacteria. Reaction conditions consisted of an initial 95°C for 3 min followed by 34 cycles of 95°C for 30 s, 55°C for 30 s, and 72°C for 45 s, and a final extension of 72°C for 10 min. To assess quality, the PCR product for each sample was subjected to electrophoresis (2% agarose, 5 v/cm, 40 min). Gels were stained with a buffer containing SYBR Gold Nucleic Acid Gel Stain (Invitrogen, USA). DNA fragments were excised from the gel and further purified using AxyPrepDNA Gel Extraction Kit (Axygen Biosciences, Union city, CA, U.S.). The concentration of DNA in the purified PCR products was further quantified using QuantiFluor^TM^-ST (Promega, USA). Amplicons were combined into two separate pooled samples (keeping bacterial and fungal amplicons separate) at roughly equal ampliﬁcation intensity ratios, and submitted to the Majorbio Bio-Pharm Technology Co., Ltd., (Shanghai, China) for sequencing on an Illumina MiSeq instrument. For biomass analysis, the PCR mixture consisted of 10 ng of genomic DNA, 0.5 μM of each primer, 10 μL of 2×SYBR Premix and water for a total volume of 20 μL respectively. For biomass of bacteria, the amplification condition consisted of an initial denaturation at 94°C for 10 s, then 40 cycles of 94°C for 5 s, 56°C for 15 s, 72°C for 15 s; and finally one cycle of 72°C for 5 min. For biomass of fungi, the amplification condition consisted of one cycle of 94°C for 45 s, then 45 cycles of 94°C for 15 s, 58°C for 15 s, 72°C for 15 s; and finally one cycle of 72°C for 5 min.

**Sequence processing and diversity analysis**

Raw Illumina fastq ﬁles were de-multiplexed, quality-ﬁltered, and analysed using QIIME (version 1.17)^1^ with the following criteria: (i) The 300-bp reads were truncated at any site that obtained an average quality score of <20 over a 10-bp sliding window, and the truncated reads shorter than 50 bp were discarded; (ii) exact barcode matching, two nucleotide mismatch in primer matching, and reads contain inambiguous characters were removed; and (iii) only overlapping sequences longer than 10 bp were assembled according to their overlapped sequence by FLASH software^2^, and reads that could not be assembled were discarded. The assembled reads were unique by dereplication and operational taxonomic units (OTUs) with a threshold of 97% pairwise identity were clustered using UPARSE (version 7.1, <http://drive5.com/uparse/>), and chimeric sequences were identified and removed using UCHIME^3^. The picked representative OTU sequences were annotated using the RDP bacterial 16S rRNA database (Release 11.1 <http://rdp.cme.msu.edu/>) and the UNITE fungal ITS database (Release 6.0 <http://unite.ut.ee/index.php>)^4^. Relative abundances of the taxa at each taxonomic level (kingdom, phylum, class, order, family and genus) were calculated and compared. The representative OTU sequences sequences were aligned using PyNAST^5^ against the template alignment and ﬁltered to remove positions which are all gaps, or not useful for phylogenetic inference. Any OTU representing less than 0.001% of the total ﬁltered sequences was removed to avoid inclusion of erroneous reads, leading to in ﬂated estimates of diversity^6^. Analysis of α-diversity and β-diversity were performed using Mothur (version v.1.30.1)^7^ with 97% pairwise identity.

**Analysis of flavours during the AAF process**

The contents of sugars, organic acids, amino acids, alcohol and volatile flavours were detected using modern analytical technologies. Before analysis, wet vinegar *Pei* was mixed with triple-distilled water in flask and filtered through Whatman paper. The filtrate was used to analyse the flavours. Sugars (fructose (Fruc), glucose (Gluc)) were analysed by high performance liquid chromatography (HPLC) (Cosmosil packed column sugar-D

(4.6×250 mm), RID). The mobile phase was acetonitrile/water at a 78:22 ratio (v/v). Organic acids (acetic acid (AA), lactic acid (LA), succinic acid (SA), oxalic acid (OA), pyruvic acid (PA), ketoglutaric acid (KgA), citric acid (CA), pyroglutamic acid (PgA) and tartaric acid (TA)) were analysed by reversed-phase HPLC (Waters Atlantis T3 column (4.6×250 mm), UVD 210 nm). The mobile phase was sodium dihydrogen phosphate at 20 mM (pH 2.7). Contents of amino acids (Glu, Asp, Ser, His, Gly, Thr, Arg, Ala, Gaba, Tyr, Cys, Val, Met, Phe, Ile, Leu, Lys, and Pro) were also determined by HPLC (Hypersil ODS column (4.6×250 mm), UVD 338 nm, 262 nm)^8^. Volatile flavours were determined by gas chromatography combined with mass spectrometry (DB-Wax capillary column, 0.32 mm×30 m). The GC oven temperature was maintained at 40^○^C for 5 min, raised 5^○^C/min to 90^○^C, and then raised to 230^○^C at 12^○^C/min and held for 8 min. The ion source and interface temperature were set at 250^○^C and 200^○^C, respectively. The mass detector was operated in the positive ion electron impact ionisation (EI+) mode at 70 eV in a range of 33–450 amu.

**Correlation analysis between microbiota and flavours during AAF**

Bidirectional orthogonal partial least squares (O2PLS) modelling was used to unveil the association between microbiota at the genus level and each flavour during AAF process. The O2PLS method is evolved from the conventional partial least squares (PLS), which consists of simultaneous projection of both the *X* and *Y* matrices on low dimensional hyper planes. In contrast to PLS, O2PLS is bidirectional (*X*↔*Y*), partitioning the systematic variability in *X* and *Y* into three parts, (i) the *X/Y* joint predictive variation, (ii) the variation in *X* orthogonal to *Y* (*X*-unique variation), and (iii) the *X*-unrelated variation in *Y* (*Y*-unique variation). The *X/Y* joint predictive variation (*Predictive component*) is the core part of O2PLS modelling, which describes the overlap information between *X* and *Y*. This part produces four vectors, score vectors for *X* (*T*), score vectors for *Y* (*U*), loading vectors for *X* (*P’*), and loading vectors for *Y* (*Q’*), in which *TP’* and *UQ’* hold the joint *X/Y* overlap information. The number of score vectors and loading vectors is determined

using cross validation. The ability to interpret the *X/Y* joint predictive variation separated from the non-correlated variation implies the model interpretation is refined and simplified. The variation in *X* orthogonal to *Y* can be further divided into two parts; one is equivalent to the component in OPLS (*Orthogonal in X (OPLS)*), which effect to achieve the best possible prediction and interpretation. The second part (*Orthogonal in X (PCA)*) consists of the structured variation that does not affect the prediction but can be interesting to further improve the interpretation of complex multivariate data. In the similar way the *X*-unrelated variation in *Y* can be divided into two parts (*Orthogonal in Y (OPLS)*, *and Orthogonal in Y (PCA)*)^9^. Variable Importance in the Projection (*VIP*) is the sum over all model dimensions of the variable influence contributions, taking into account the influence of every term in *X* on all the *Y* variables. For O2PLS method, there are three *VIP* vectors to compute; *VIP(pred)*, *VIP(orth)*, and *VIP(tot)*, which represent *VIP* value for the predictive components, the orthogonal components and both predictive and orthogonal parts respectively. Terms with larger *VIP* value, larger than 1.0, are the most relevant for explaining *Y* variables. The correlation matrix shows the pair-wise pearson correlation between all variables (*X* and *Y*), representing the extent of the linear association between the two terms.

**Supplementary references**

1. Caporaso, J. G. *et al.* QIIME allows analysis of high-throughput community sequencing data. *Nat. Methods* **7,** 335-336 (2010).

2. Magoč, T. & Salzberg, S. L. FLASH: fast length adjustment of short reads to improve genome assemblies. *Bioinformatics* **27,** 2957-2963 (2011).

3. Edgar, R. C., Haas, B. J., Clemente, J. C., Quince, C. & Knight, R. UCHIME improves sensitivity and speed of chimera detection. *Bioinformatics* **27,** 2194-2200 (2011).

4. Abarenkov, K. *et al.* The UNITE database for molecular identification of fungi –recent updates and future perspectives. *New Phytol.* **186,** 281-285 (2010).

5. Caporaso, J. G. *et al.* PyNAST: a flexible tool for aligning sequences to a template

alignment. *Bioinformatics* **26,** 266-267 (2010).

6. Bokulich, N. A. *et al.* Quality-filtering vastly improves diversity estimates from Illumina amplicon sequencing. *Nat Meth.* **10,** 57-59 (2013).

7. Schloss, P. D. *et al.* Introducing mothur: open-source, platform-independent, community-supported software for describing and comparing microbial communities. *Appl. Environ. Microbiol.* **75,** 7537-7541 (2009).

8. Versari, A., Parpinello, G. P., Mattioli, A. U. & Galassi, S. Characterisation of Italian commercial apricot juices by high-performance liquid chromatography analysis and multivariate analysis. *Food Chem.* **108,** 334-340 (2008).

9. Trygg, J. O2-PLS for qualitative and quantitative analysis in multivariate calibration. *J. Chemometrics* **16,** 283-293 (2002).
